# Anharmonic Thermal Motion Modelling in the Experimental XRD Charge Density Determination of 1-Methyluracil at T = 23 K

**DOI:** 10.3390/molecules26113075

**Published:** 2021-05-21

**Authors:** Riccardo Destro, Pietro Roversi, Mario Barzaghi, Leonardo Lo Presti

**Affiliations:** 1 Chemistry Department, Università degli Studi di Milano, Via Golgi 19, 20133 Milano, Italy; leonardo.lopresti@unimi.it; 2 Department of Molecular and Cell Biology, Leicester Institute of Structural and Chemical Biology, University of Leicester, Lancaster Road, Leicester LE1 7HB, UK; pr159@leicester.ac.uk; 3 Consiglio Nazionale delle Ricerche (CNR), Piazzale Aldo Moro 7, 00185 Roma, Italy; mario.barzaghi@cnr.it; 4 Istituto Nazionale di Fisica Nucleare (INFN), Laboratori Nazionali di Frascati, 00044 Frascati, Italy

**Keywords:** 1-methyluracil, X-ray diffraction, charge density, electrostatic moments, quantum theory of atoms in molecules, anharmonicity, quantum chemistry, molecular dynamics, crystal chemistry

## Abstract

The experimental electron density distribution (EDD) of 1-methyluracil (1-MUR) was obtained by single crystal X-ray diffraction (XRD) experiments at 23 K. Four different structural models fitting an extensive set of XRD data to a resolution of (sinθ/λ)_max_ = 1.143 Å^−1^ are compared. Two of the models include anharmonic temperature factors, whose inclusion is supported by the Hamilton test at a 99.95% level of confidence. Positive Fourier residuals up to 0.5 eÅ^–3^ in magnitude were found close to the methyl group and in the region of hydrogen bonds. Residual density analysis (RDA) and molecular dynamics simulations in the solid-state demonstrate that these residuals can be likely attributed to unresolved disorder, possibly dynamical and long–range in nature. Atomic volumes and charges, molecular moments up to hexadecapoles, as well as maps of the molecular electrostatic potential were obtained from distributed multipole analysis of the EDD. The derived electrostatic properties neither depend on the details of the multipole model, nor are significantly affected by the explicit inclusion of anharmonicity in the least–squares model. The distribution of atomic charges in 1-MUR is not affected by the crystal environment in a significant way. The quality of experimental findings is discussed in light of in-crystal and gas-phase quantum simulations.

## 1. Introduction

**Figure 1:**
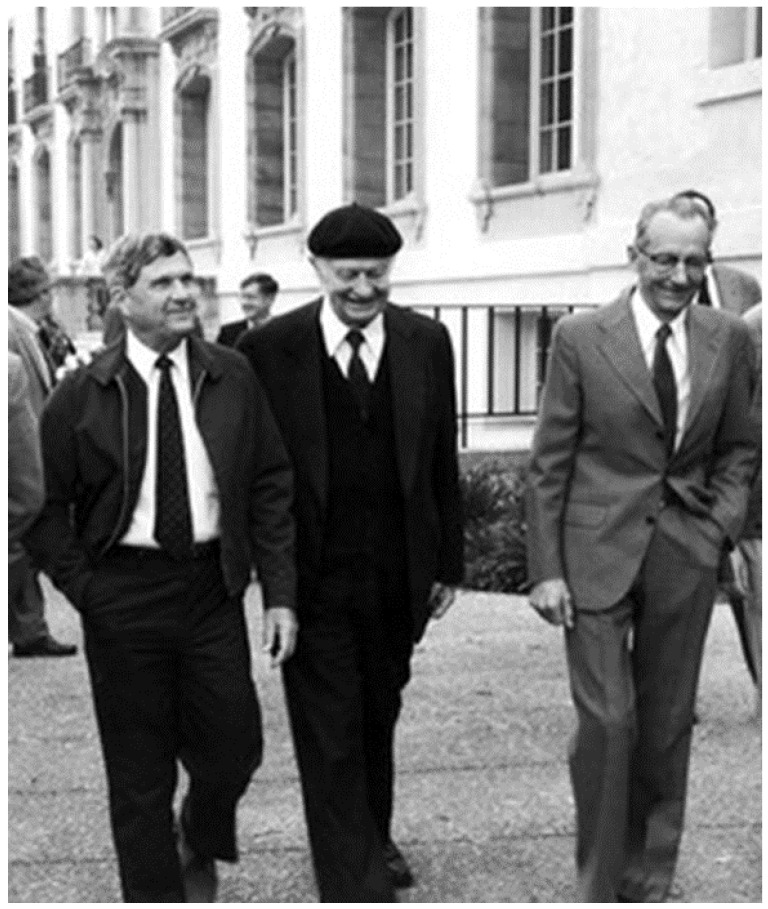
Linus Pauling at the California Institute of Technology during his visit on his 85th birthday in 1986 with Dick Marsh on his right and Sten Samson on his left. © Courtesy of Caltech archives (ID #PR-86-050-14D-25). Reproduced with permission.

“*I think that it is very interesting that one can see the functions of Schrödinger’s wave mechanics by means of the X-ray study of crystals. This work should be continued experimentally. I believe that much information regarding the nature of the chemical bond will result from it*.” This statement, written in 1926 by a young Linus Pauling to A. A. Noyes, the head of the chemistry division at Caltech, could not have been more prophetic. It took more than 35 years, but the remarkable hardware development in the second half of the 20th century allowed solid state scientists to access experimental charge density properties of an impressive number of organic and inorganic compounds [1], paving the way to the modern research in molecular recognition and self–assembling. In this respect, of great importance was the work of those who pushed the limit of what was thought possible at the time young Pauling was writing to A.A. Noyes. Among them, Richard Marsh and Sten Samson (Figure 1) pioneered the application of advanced computing and cryogenic techniques to X–ray crystallography. 

Following their footsteps on the path they traced, we here present the experimental charge density analysis of 1-methyluracil (hereinafter 1-MUR), based on a very low-T dataset collected at Caltech in Marsh’s laboratory in 1989 by one of us (RD). Experimental as well as theoretical and computational work on 1-methyluracil and its derivatives have been published a number of times [2,3,4,5,6,7], yet the experimental estimates for some of the fundamental properties of the molecule in a condensed phase, such as integrated atomic volumes and charges, as well as atomic and molecular electrostatic quadrupole moments, are not available, to the best of our knowledge, in the literature.

One of the best means for obtaining reliable values for these quantities is the analysis of crystalline X-ray diffraction (XRD) charge densities, ρ (**r**), according to QTAIM, the theory of atoms in molecules due to Richard Bader [8]. This theory, originally devised for theoretical ρ (**r**)s, is now widely applied to experimental electron density distributions (EDDs) as well, with optimal results when ρ (**r**)_exp_ is derived from very accurate, high resolution, and possibly very low temperature (LT) diffracted data sets.

In the field of experimental XRD charge density analysis, appropriate modelling of the H atoms is essential, as is an adequate modelling of the anisotropic displacement parameters (ADPs) of all atoms. A comparative study has recently examined [9] some of the most frequently adopted strategies to obtain H positions and ADPs and has concluded that the Hirshfeld atom refinement (HAR) method yields the most accurate C–H bond distances compared with neutron data results. The HAR has been classed [10] as “*a novel X-ray structure refinement technique that employs aspherical atomic scattering factors obtained from stockholder partitioning of a theoretically determined tailor-made static electron density*”, and its accuracy and precision have been validated [10,11,12]. However, various other methods [13,14,15,16,17,18] have been devised for a proper treatment of hydrogen atoms in single-crystal XRD investigations, and all of them, with the exception of the “polarized H atom case” implemented in the program VALRAY [18], include an estimation of hydrogen atoms’ ADPs. 

As for the anharmonicity of thermal motion, the relevance of its inclusion in ADP models when analyzing EDDs in crystals is still an open question, the uncertainty being partly related to the fact that very asphericity in atomic charge densities can be due to genuine anharmonic thermal motion and/or to chemical bonding [19], as well as to static or dynamic disorder [20,21]. Long ago, at an international symposium on the accuracy in structure factor measurement, Werner F. Kuhs presented [22] a review on the properties of the most widely used formalisms to describe anharmonicity in crystallographic structure analysis and later published a paper [23] on both the theoretical and experimental aspects of generalized ADPs. In both works, the author reported an equation for calculating the minimum experimental data resolution required to include into the temperature factor a significant Gram–Charlier expansion of the harmonic displacements. Philip Coppens and coworkers had shown [24] that in accurate X-ray diffraction studies an approximate separation between aspherical charge-density effects and anharmonic motion is feasible, although the two corresponding formalisms are often equally efficient in accounting for the observations on their own. Although in many cases the identification and separation of the two effects may be inaccurate or even impossible [19], in a few cases only a combined Gram–Charlier multipole refinement [25,26] has avoided erroneous interpretations of the charge density analysis. 

The effects of neglecting anharmonic nuclear motion in charge density investigations, when anharmonicity is definitely present, have been studied [27] based on *model* structure factors. Several statistics were examined, from the crystallographic R value to the thermal motion and the density parameters, with a main focus on the effects on the residual density distribution and the EDD topology but without analysis of the influence of anharmonic motion modelling on integrated atomic charges, volumes, or electrostatic potential and electric moments. Similarly, a detailed charge density investigation based on experimental X-ray data [28] has proven the need to include anharmonicity into the thermal motion, even for data collected at 15 K, to flatten the residual density map, and has focused on the refined multipole parameters (“*distorted when the anharmonic motion was not properly refined*”), but no report was made on the influence of anharmonicity on the electrostatic properties. 

A suitable system for such investigation is crystalline 1-MUR (C_5_H_6_N_2_O_2_): all atoms of the molecules (except for two mirror-related methyl H atoms) lie on the mirror plane of the crystal in space group Ibam. This 1-MUR crystal form was investigated several years ago by neutron diffraction [29] at T = 15, 60 and 123 K; and by XRD, with data collected [30] both at T = 123 K and at a temperature probably differing, in the words of the authors, by about 20 K from the intended 123 K. That X-ray investigation—unlike the neutron study [29]-was not devoted to gain a better understanding of the thermal vibrations in the crystal. Rather, the aim was to retrieve electrostatic properties, but the limited data extension (sinθ/λ < 0.69 Å^−1^) prevented an adequate parametrization. As for the X-ray data of ref. [30], which extended to sinθ/λ < 1.08 Å^−1^, four different refinement models were tested, but none included anharmonic corrections to the ADPs. That X-ray investigation retrieved electrostatic properties from the diffraction data, although the data quality was not the best, as revealed by inconsistencies in the cell parameters with respect to the values of the neutron investigation and by anomalies in the U_33_’s of all nine non-H atoms (the 1-MUR molecular plane is orthogonal to the c axis of the crystals).

In the present work, we compare results from four different multipolar refinements of XRD data collected from a prismatic crystal of 1-MUR at T = 23 K and (sinθ)/λ up to 1.143 Å^−1^. Two of the four multipole models include anharmonic temperature factors through Gram–Charlier modifications of the ADPs. Comparison of the statistical significance of the crystallographic R values is made using the Hamilton significance test [31], while two distinct distributed multipole analyses (DMA), called Stewart DMA and QTAIM DMA, implemented in the Properties of Atoms and Molecules in molecular Crystals (PAMoC) suite of programs [32] are used to evaluate the atomic and molecular electrostatic moments resulting from the four refinements. We also compare the molecular electrostatic potential (as calculated with the VALRAY2000 program [18]) and the integrated atomic volumes and charges as calculated with PAMoC from the experimental ρ (**r**). 

## 2. Experimental

**Figure 2:**
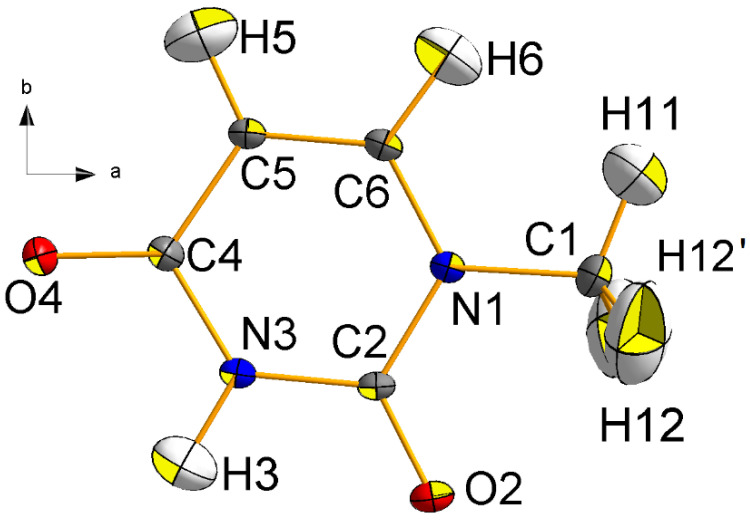
Central projection of 1-methyluracil (1-MUR) at T = 23 K in the mirror (*a*,*b*) plane, as seen along the *c* cell axis. The atom numbering scheme and the crystallographic reference system are also shown. Thermal ellipsoids are drawn at the 90% probability level. O: red; N: blue; C: grey; H: white.

**Figure 3:**
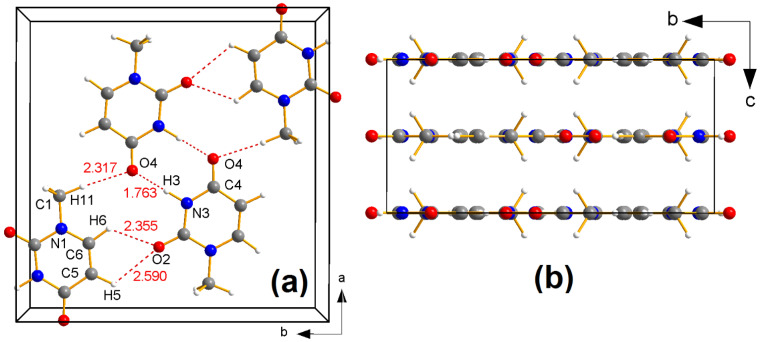
(**a**) Central projection of crystal packing of 1-MUR in the (*a*,*b*) plane at T = 23 K, as described in previous studies [29,30], down the *c* axis. Relevant hydrogen-bonded contacts are shown as dashed red lines, with the corresponding distances in Å. (**b**) Parallel projection of the 1-methyluracil unit cell along the *a* axis. Atoms colored as in Figure 2.

The crystal was a gift of the late professor Bryan Craven. A plot of the asymmetric unit and the atomic numbering scheme is shown in Figure 2. All atoms but H12 lie on a mirror plane. Crystal packing (in agreement with two previous studies [29,30]) is illustrated in Figure 3.

In most of our XRD investigations, samples [33] for accurate and precise charge density studies are machined to approximate spherical shape, but in the 1-MUR case no mechanical rounding of the crystal was possible because every cut, or even a slight pressure on some of the faces, causes an unacceptable degradation of crystal quality: this is likely due to the layered crystal packing (Figure 3b), which makes the crystals easily flake along planes parallel to (*a*,*b*), on which the molecules lie. 

Diffraction data were measured at the X-ray Facility of the California Institute of Technology in Pasadena (US). The sample, glued on the tip of a glass capillary, was mounted on a four-circle diffractometer equipped with the prototype of the Samson cryostat [34] and a conventional point detector. Figure S1 and Table S1 in the Supplementary Materials report on the cell edges as a function of T. Upon cooling, the crystal undergoes significant anisotropic distortion: the overall reduction of the cell volume is dominated by a ~3.5% shrinking of the *c* axis, while the *a* cell axis shows a very small (0.1%) but detectable thermal contraction. The temperature was stable at 22.9 ± 0.7 K throughout data collection. The X-ray diffraction experiment followed the procedure described in detail in published reports of our earlier low-temperature experimental charge density studies of other crystals [35,36,37]. After data collection, low-angle data (2θ_Mo_ < 20°) were re-measured at a lower current setting to minimize possible problems associated with nonlinearity of the photon counting system. All intensity measurements were corrected for scan-truncation losses according to an empirical method [38,39,40] based on profile analyses and background distributions as a function of 2θ. Corrections were made for Lorentz and polarization effects but not for absorption (the latter was assumed to be negligible). A summary of crystal data and details of data collection is given in Table 1.

### Multipolar Refinement of Four Models

Early processing of the Caltech data described above, with a VALRAY2000 least-squares refinement of an independent atom model (IAM), resulted in Fourier difference maps with unusually high residuals, particularly in the proximity of the methyl group. To check if this was due to undetected LT technical problems, the same crystal was mounted on a second diffractometer (in Milan (Italy))—equipped with a local version of the Samson cryostat—and data collection was repeated at T = 18–20 K, leading to a set of 8952 intensity measurements. After treatment for scan-truncation errors and merging of the equivalent reflections, an IAM least-squares refinement gave Fourier difference maps very similar to those previously obtained from the Pasadena data, with a peak of ~0.6 eÅ^−3^ near the methyl group and another peak, ~ 0.45 eÅ^−3^ high, in proximity of the shortest intermolecular hydrogen bond. Additional X-ray diffraction data were then measured (also in Milan) from three other 1-MUR crystals of different sizes and shapes, both at RT and at T = 18–24 K. 

All residual maps invariably showed the same features, with peak heights obviously depending on the data resolution (one of the samples was rather small, 0.20 × 0.075 × 0.05 mm and was used mainly to investigate the effects of extinction, very relevant for reflection 002). It was concluded that the observed features of the maps were related to some intrinsic properties of the 1-MUR crystals. One of these possible properties was anharmonic thermal motion, which prompted us to investigate the crystal structure using four different models (labelled A–D, see next paragraph). For the full-matrix least-squares refinement of the 1-MUR low-T structure, multipolar scattering factors were used [41,42,43], as implemented in the VALRAY2000 program [18], to model atomic EDD asphericity. The corresponding pseudo-atom model A included 179 parameters: an extinction coefficient; coordinates and *U*_ij_s for the nine non-H atoms (for a total of 18 + 36 parameters, owing to the symmetry restrictions); for each non-H atom—one core monopole term, constrained to have the same value for all the nine atoms, plus one monopole, two dipolar, three quadrupolar, and four octupolar population coefficients; and multipolar coefficients up to the quadrupole level for the H atoms. Positional parameters of the latter atoms were those of the low-T neutron work [29], while for their anisotropic *U*_ij_s, the values of the model B in Table 2 of reference [17] were used. Both coordinates and *U*_ij_s of the H atoms were not further refined. No scaling factors were applied to these *U*_ij_s with respect to the neutron estimates [29]. We checked carefully, though, that by switching positional and thermal parameters between X-rays and neutrons (e.g., by using both xyz and *U*_ij_ from neutrons) the electronic and electrostatic properties here discussed remain statistically identical.

In model B, the 45 symmetry-allowed hexadecapole terms of the non-H atoms were added to the previous set of parameters, while model C was an extension of model B with the inclusion of the 88 non-vanishing third-order Gram–Charlier (GC) coefficients (*C_ijk_*s) for all 14 symmetry-independent atoms. Finally, model D was created by adding to model C the 81 allowed fourth-order GC coefficients (*D_ijkl_*s) of the nine non-H atoms. In addition to positional, thermal, and multipolar parameters, according also to Klooster et al. [30], all models refine a Becker–Coppens secondary isotropic extinction coefficient [44,45] of type 1 with a Lorentz distribution of the mosaic blocks (g_11_ in VALRAY2000). Extinction is large for (0 0 2) (Y_ext_ = 0.65) and somewhat lower, yet highly significant (0.902 ≤ Y_ext_ ≤ 0.948) for (0 0 4), (2 0 0), (1 2 1), (1 1 0), and (2 4 0). Final values for the extinction coefficient are 0.527 (9) × 10^–4^ rad^–1^ for Model A and 0.529(9) × 10^–4^ rad^–1^ for Model D. These correspond to a full width at half maximum of the angular spread of crystallites as large as 6.04 × 10^–5^ rad = 12.5” arc (Model A) and 6.02 × 10^–5^ rad = 12.4” arc (Model D). In conclusion, the refined extinction model does not depend on the details of the multipole model. The results of the multipolar least-squares refinements are reported in Table 2.

The analysis of the experimental ρ (**r**) in terms of topological features, nuclear-centered distributed multipole analyses (DMA) and derived electrostatic properties was carried out both with the VALRAY [18] and PAMoC programs [32]. Residual density analysis was carried out with jnk2RDA v1.5 [46]. CCDC entry 2077825 contains the supplementary crystallographic data for this paper. These data can be obtained free of charge from the Cambridge Crystallographic Data Centre via www.ccdc.cam.ac.uk/structures.

## 3. Results and Discussion

### 3.1. Hamilton’s Test

Well aware of the limited resolution of our data set, rather short of Kuhs’ minimum rule [22,23], we nevertheless decided to investigate the possibility of including anharmonic terms in the thermal motion parameterization of our crystal at T = 23 K—using the Hamilton test as an adequacy criterion. It was in fact on the basis of this test that the authors of the neutron study of 1-MUR [29] deemed anharmonic treatment unwarranted because none of the resulting small reductions in agreement factors was significant at the 99.5% confidence level. Similarly, the Hamilton test was used by Stewart, Larsen, and coworkers [25] to choose the best anharmonicity model in a charge-density study of tetrafluoroterephthalonitrile, where third-order GC coefficients were applied only to the nitrogen atom because a test refinement with anharmonic motion on all five independent atoms could be rejected at the 50% level of confidence.

We applied this test to the results of the least-squares refinements of the 1-MUR structure. For each of the three models B, C, and D, the significance of the reduction of the residual ΣwΔ^2^ due to the inclusion of extra parameters (see Table 2) was estimated, and in all three cases the improvement of the fit was significant at the 99.95% level of confidence. Details of the calculations, referring to the 3344 observed data, are reported in Table 3. The significance points of ℛ_b,n-m_ were obtained according to Equation (24) of reference [31], that is ℛ_b,n-m,α_ = {[b/(n-m)] × F_b,n-m,α_ + 1}^½^, with the F values taken from the web [47]. 

### 3.2. Analysis of Charge Density Residuals

**Figure 4:**
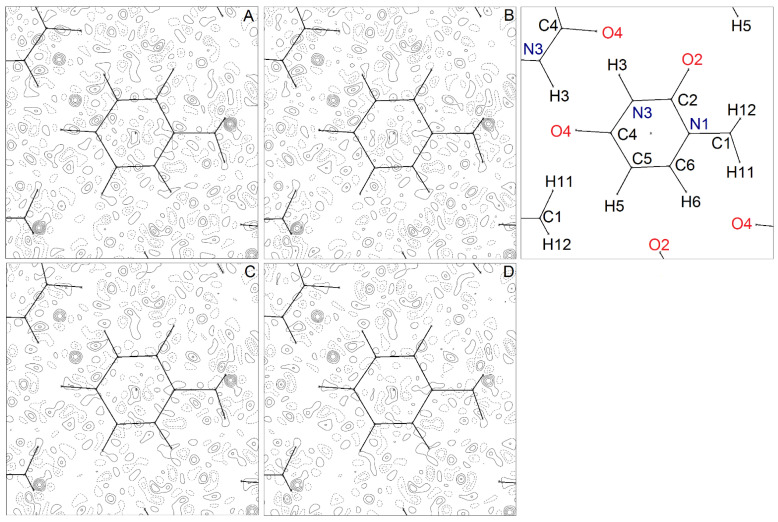
Residual Fourier density map, $\Delta \rho =1/V\sum_{hkl}\left(F_{hkl}^{o}-F_{hkl}^{c}\right)∙exp\left[-2\pi i\left(hx+ky+lz\right)\right]$, for 1-MUR at T = 23 (1) K, as computed for the multipole models (**A**–**D**), where $F_{hkl}^{o}$ and $F_{hkl}^{c}$ are observed and calculated structure factor amplitudes (on the same scale), and *V* is the cell volume. All plotted atoms lie in the (*a*,*b*) plane, with the only exception of H12 and its mirror image, which lie ± 0.9 Å above and below it, respectively. The atom numbering scheme is shown in the upper right box. The map is 9 × 9 Å wide and is plotted in the (*a*,*b*) plane. The origin is at the center of the 6-membered ring. Contour levels are drawn at steps of 0.1 eÅ^–3^ as full (dashed) lines if positive (negative) and range from −0.4 eÅ^−3^ to +0.6 eÅ^−3^. The zero contour is omitted for the sake of clarity. Maximum and minimum residual densities (in eÅ^−3^) are +0.67/−0.31 (**A**), +0.60/−0.36 (**B**), +0.57/−0.32 (**C**), and +0.49/−0.37 (**D**).

A further test of the accuracy of each multipolar model resides in the quality of the fit to the experimental diffraction pattern. Figure 4 compares the residual density Fourier maps obtained with our models A–D, highlighting the experimental signal that the multipole charge density models are not able to account for. If not otherwise specified, the analysis was carried out for the whole dataset of 3344 independent reflections with I > 0.

Apart from the exceptions detailed below, all residual maps are very similar and generally featureless (Figure 4). In Model A, some peaks, with magnitude up to +0.2 eÅ^−3^, appear close to some covalent bond midpoints in the 6-membered ring system. These positive residuals are mainly due to local high-order aspherical features of the charge density, and indeed they are considerably reduced in Model B, where the expansion includes *l* = 4 poles on non-H atoms. Further addition of high-order cumulants makes these residuals completely disappear (Figure 4, panels C and D), corroborating the hypothesis that some residual anharmonicity is still present in 1-MUR well below liquid nitrogen temperature. 

In all maps, two significant positive peaks of Δρ are clearly detectable. The first one lies in the hydrogen bond region (upper left of the plot, Figure 4A–D): it has a spherical shape, and its magnitude (~ 0.4 eÅ^−3^) is independent of the multipole model. The second one is close to the H12 methyl hydrogen: it has a more structured shape, and its magnitude undergoes a ~27% reduction on going from Model A to Model D (Figure 4). Both residual peaks survive the introduction of third- and fourth-order Gram–Charlier cumulants in the least-squares models. Thus, their origin remains to be explained. We note that these residuals are not specifically due to weak reflections, as they are still perfectly recognizable (though significantly reduced) if the Fourier maps are computed just on the 2614 data with I > 3σ (I) (Figure S3 Supplementary Materials). Adoption, for the *U*_ij_s of the H atoms, of the values obtained in the neutron diffraction work at T = 15 K [29] instead of those we estimated from the rigid body analysis of non-H atoms ADPs at T = 23 K [17] did not significantly change positions of these Δρ maps minima and maxima nor did it change their values. 

To gain insights into the nature of such features, a residual density analysis (RDA) was carried out according to Henn and Meindl [46,48]. The full discussion can be found in the Supplementary Information (Section S2); here we summarize the main results. 

The RDA analysis shows that the data are slightly overfitted; that is, some noise is absorbed by the density model. Moreover, EDD residuals persist even after the addition of higher order cumulants (Model D). These manifest in a shoulder of the fractal dimension plot for Δ*ρ* > 0 residuals (Figure S2 Supplementary Materials) and are clearly related to the large Fourier residuals in the main molecular plane (Figure 4). Moreover, such features do not depend on either weak (I < 3σ (I)) reflections (Table S3, Figures S3 and S4 Supplementary Materials) or noise levels (Table S4, Figure S5 Supplementary Materials). Rather, as these residuals are present in all the specimens examined, irrespective of the model adopted, they likely have a genuine physical origin, which is not accounted for by any of our models A–D. 

### 3.3. Molecular Dynamics 

To shed light on the possible nature of our models’ inability to account for the observed residual features, we performed classical molecular dynamics (MD) simulations on crystalline 1-MUR with the MiCMoS package [49,50,51,52]. Full details of the procedure are documented in Section S3 Supplementary Materials.

**Figure 5:**
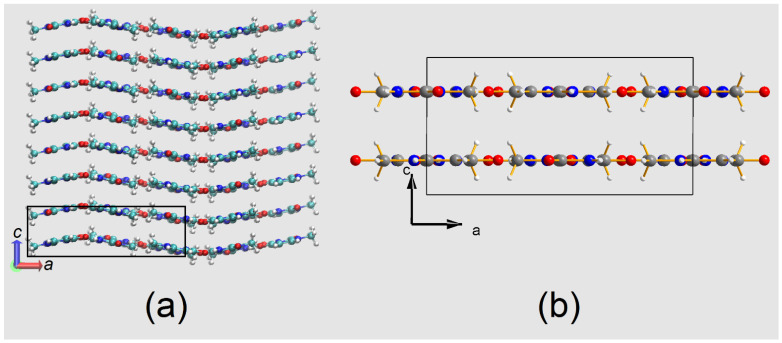
(**a**) Mean MD simulation box, as obtained from the time average of frames over the last 300 ps of the trajectory. The box contains 2 × 2 × 4 crystallographic unit cells, one of which is highlighted as a black rectangle. (**b**) Mean MD crystallographic unit cell, as obtained by the space-average of the simulation box in (**a**). Apart from a shift of the origin by ¼ *c*, it is comparable with the experimental one shown in Figure 3b. Both views are down the *b* axis.

The mean MD simulation box, averaged over the last 300 ps of the trajectory at T = 23 K, is shown in Figure 5a. A slight commensurate modulation of the molecular orientation across the simulation box is appreciable along the *a* axis, with wavelength of ≈25 Å and amplitude not exceeding ±2.5 Å, i.e., roughly the thickness of one molecular layer. This arrangement allows the system to gain packing energy by alleviating the atom–atom repulsion due to close contacts at low T. At the same time, this modulation produces a slanted column motif, which maximizes dispersive interactions. [53] If molecules in the simulation box are back-translated into the crystallographic cell and space-averaged, the experimental crystal packing is recovered (Figure 5b), as the phases of long-range oscillations from different regions of the supercell cancel out. In other words, the perfect (*a*,*b*) mirror symmetry of the Ibam space group is lost when the long-range disorder is considered. This is not uncommon: for example, in the centrosymmetric chloroquine dihydrogen phosphate salt, the inversion center requires that some co-crystallized water molecules produce a frustrated (disordered) H-bond pattern. [54] We do not claim that our MD model represents the correct long-range structure of 1-MUR, as the predicted amplitude and wavelength of the modulation may depend on truncation effects and details of the force field. However, the simulation points out that some deviations from the planarity of the packing motif imposed by the crystal symmetry are possible in this structure when dynamic effects are explicitly considered. The modulation survives when larger simulation boxes are employed (Figure S7 Supplementary Materials) and when there is a very good linear correlation among the MD-predicted thermal parameters and the experimental ones (Figure S8 Supplementary Materials), corroborating the general validity of our approach. Based on these results, the persistence of the aforementioned large Fourier residuals and the significant amount of noise in the dataset are likely underpinned by unresolved disorder, possibly dynamic in nature. The MD prediction awaits further experimental confirmation, but, as we are going to demonstrate in the next Sections, the accuracy of the multipole analysis is still good enough to extract high-quality chemical information.

### 3.4. The Positional Parameters

A close inspection of the X and Y coordinates of the nine non-H atoms (Table S8) reveals the following relevant features: (i) the individual values of model A are practically identical to those of model B, with differences never exceeding 1 estimated standard deviation (esd); (ii) insertion of third-order GC coefficients (model C) implies an increase of the esds by 2.5 to 5 times, and larger differences between corresponding parameters are now observed, up to 4.8 esds (of model C) for the X coordinate of atom O4; (iii) the parameters of model D do not differ from those of model C by more than 2 esds, the latter quantities showing similar values in the two models; (iv) these large standard deviations are similar and often slightly less than those of the neutron diffraction study at T = 15 K, whose derived coordinates are in close agreement with those of models A and B, with the possible exception of the X parameter of atom C1, differing by 4.3 times the neutron esd; and (v) the same coordinate shows the greatest variation across the five sets of positional parameters in the table, with the model D value differing from the neutron value by 7.3 esds.

The large increase of the esds after insertion of third-order cumulants, i.e., upon going from model B to model C, is related to the relatively high correlation coefficients, with values in the range 0.843–0.894, between all X and Y coordinates and third-order GC coefficients in the least-squares refinement of model C. 

An obvious effect of the increased coordinate uncertainties is the corresponding increase of the esds of the bond distances and of the intermolecular contacts. This is shown in Table S9 Supplementary Materials in the supplementary material: for each model and from the 15K neutron diffraction study, the Table lists the length values of the nine bonds between the non-H atoms and some relevant intermolecular separations. For the intramolecular bonds, the esds of the models C and D, all equal to 0.0009 Å, are three times those of models A and B, yet still less than the esds of the neutron work, 0.001 Å. The largest difference between the XRD mean values and the neutron estimates occurs at the C1–N1 bond and amounts to 0.004 Å. All the other differences between the arithmetic averages and the neutron values are within 0.002 Å, which is also the difference for the N3...O4’ H-bond length. As for the intermolecular contacts involving H atoms reported in the table, they all agree within 0.004 Å, which is only twice the esd of the corresponding separations resulting from the neutron study. We may conclude that the inclusion of GC cumulants in the multipolar models has little, if not a negligible, effect on the geometry of this crystal structure.

### 3.5. The Anharmonicity Gram–Charlier Coefficients

A list of the anharmonic GC coefficients of models C and D is presented in Table S10 Supplementary Materials as part of the supplementary material. Inspection of the 88 third-order cumulants inserted in model C reveals that for all nine non-H atoms except C4, at least one coefficient is >3 esds (up to five times at atoms N1 and up to 5–6 times at the two O atoms), while for the H atoms this occurs at the methyl atoms H11 and H12 and at atom H6. Four out of the nine non-vanishing fourth-order coefficients of atom C1 are greater than 3 to 7 times their esds, possibly indicating that anharmonicity particularly affects the methyl group. Of the other eight non-H atoms, C2, C5, and N3 also show one fourth-order GC coefficient with a value >3 esds.

### 3.6. The Nuclear Anisotropic Thermal Parameters 

A comparison of the values of the *U*_ij_s obtained from the refinement of the four models (see Table S8 Supplementary Materials) shows that the addition of hexadecapoles for C, N, and O atoms to model A, as well as the insertion of third-order GC coefficients to model B, do not affect the *U*_ij_s. Only a few of these parameters differ at the most by one esd between models A and B. The introduction of 81 fourth-order GC coefficients in model C also causes modest variations: the most remarkable change is the increase, by 5–7 times, of the esds, due to the large correlation of all new anharmonic parameters with the *U*_ij_s, with correlation coefficients in the range of 0.824–0.957. This effect is not unexpected and has been described previously [20,55].

### 3.7. Atomic Charges and Volumes

The electron population coefficients obtained from the least-squares refinements of the four multipolar models were used to generate experimental EDDs. Bader’s QTAIM [7] was applied to partition these experimental EDDs into atomic basins (Ω), and hence obtain, by integration, atomic volumes and charges. The calculations were made with the PAMoC code [32]. The results are reported in Table 4 and in Figure S9 Supplementary Materials. It is remarkable that the coefficient of variation (or relative standard deviation, CV = esd/mean) is equal to 0.002 for the molecular volume and varies from 0.003 (C6) up to a maximum of 0.031 (H3) for the atomic volumes. Similarly, CV values of atomic charges are between 0.02 and 0.07 (except for carbon atoms C1 and C5, which have slightly negative charges, oscillating between −0.012 and −0.059 e, with a CV of 0.5). From these results (see Figure S9 Supplementary Materials), it can be deduced that the inclusion of anharmonicity in the multipolar expansion does not significantly affect the values of the EDD-derived electrostatic properties.

The molecular volumes obtained by adding the atomic volumes are shown in the last row of Table 4. They are virtually the same, with a coefficient of variation equal to 0.02, and their average value, when multiplied by *Z* = 8, reproduces the unit cell experimental volume to within 0.06% (see Table 1). The quantitative accuracy of the latter estimate is comparable with those obtained in previous molecular crystals EDD studies [56,57,58].

Noteworthy are the values of atom H3, which is the H atom participating in the hydrogen-bond forming a dimer through an inversion center. The mean charge of this atom from the four models is about 3.4 times that of the other three H atoms, and the average volume value is about 2.8 times smaller than the corresponding volumes. Even larger integrated charges (0.69 and 0.66 *e*) and smaller volumes (1.0 and 1.5 Å^3^) were obtained by Flensburg and Madsen [59] with their Ω integration procedure applied to the experimental charge density of a compound with a very short O–H–O hydrogen bond [60].

In QTAIM, an atomic basin is defined as the portion of space including the nucleus and bounded by a surface *S* of local zero flux in the gradient vector of the electron density. The atomic surface *S* is the union of some interatomic surfaces, one for each bonded neighbor. Usually, for the atoms of a crystal, the surface *S* is closed, but if we imagine extracting a molecule from the crystal, portions of atomic basins can be at an infinite distance from the nuclear attractor. Therefore, these open portions of the atomic surface are replaced with an envelope of the electron density with a constant value. This procedure leads to the definition of the size and volume of an isolated molecule. The results of its application to the experimental EDD of 1-MUR are reported in Table S11 Supplementary Materials for the four experimental models and the theoretical one. The arithmetic means of the values from the four experimental models applied to both the molecule in crystal (Table 4) and the molecule extracted from the crystal (Table S11 Supplementary Materials) are graphically compared in Figure S10 Supplementary Materials. While the atomic charges remain unaffected, the volume of the molecule, after extraction from the crystal, increases by 4.1 Å^3^ (3.06%), thanks mainly to the contribution of the hydrogen atom H3 (1.56 Å^3^, i.e., 38% of the total volume increase, see Figure S10 Supplementary Materials). Only the carbon atoms C1 and C5 and the hydrogen atoms H5 and H12 undergo an overall volume contraction of 3.41 Å^3^, while atomic charges remain unchanged. The volume of the molecule isolated from the crystal (138.1 Å^3^) can be compared with the value of 146.0 Å^3^ calculated at the B3LYP/6-311G (d,p) theoretical level for a molecule whose geometry is the same as in the crystal. The latter volume does not change significantly (146.6 Å^3^) when the molecule is allowed to relax to its equilibrium geometry.

### 3.8. Molecular Electrostatic Moments

The distributed multipole analysis (DMA) based on the population parameters of Stewart’s pseudoatoms (as obtained from each least-squares structure refinement), and hence called “Stewart’s DMA”, was used in PAMoC to derive the components of the 1-MUR dipole vectors and those of the tensors, from quadrupole to hexadecapole, for each of the higher order multipole tensors. The results are reported in Table 5, and the weighted means of the values of the four models are graphically visualized in Figure S11 Supplementary Materials against theoretical B3LYP/6-311G (d,p) values, with which they correlate fairly well.

The 1-MUR molecular, dipole moment is the same in all four cases, and the average value, 5.8 ± 0.2 D, lies in between the two values, 4.4 ± 2.2 and 6.4 ± 2.7 D, given by Spackman (in SI units) for 1-MUR in his review of molecular electric moments from X-ray diffraction data [62]. The two quoted values were taken from reference [30] and come from two different refinements of XRD 123 K data. They agree with each other and with the value of 4.2 ± 0.3 D obtained in dioxane solution [63] within the rather large errors reported. For the sake of comparison, two different quantum chemical pieces of software in conjunction with different levels of theory were used to check the robustness of the predicted properties. Values for the molecular dipole moment computed for the gas-phase molecule (crystal geometry) at the MP2/6–31G* level of theory with the Gaussian16 program [64] and at the B3LYP/6-311G (d,p) level of theory with the ORCA program [61] read 4.68 and 4.90 D, respectively. Identity within fractions of the pooled standard deviation is also shown by the components of the quadrupole tensor, as well as by those of the other two tensors, whose values are never quoted, to our knowledge, in published reports of 1-MUR electrostatic properties.

As pointed out in our previous work [58,65], Stewart atoms extend to infinity and overlap with each other in the same way as in other so-called “fuzzy” partitionings of the EDD (e.g., Becke [66] and Hirshfeld’s stockholder [67,68] schemes). As fuzzy molecules have boundaries at infinity, the moments reported in Table 5 refer to a molecule removed from the crystal and are directly comparable with the theoretically calculated values. The latter therefore appear to underestimate the dipole moment values derived from Stewart’s DMA. QTAIM discrete partitioning of periodic EDDs may give two different results depending on the definition of molecular boundaries. 

**Figure 6:**
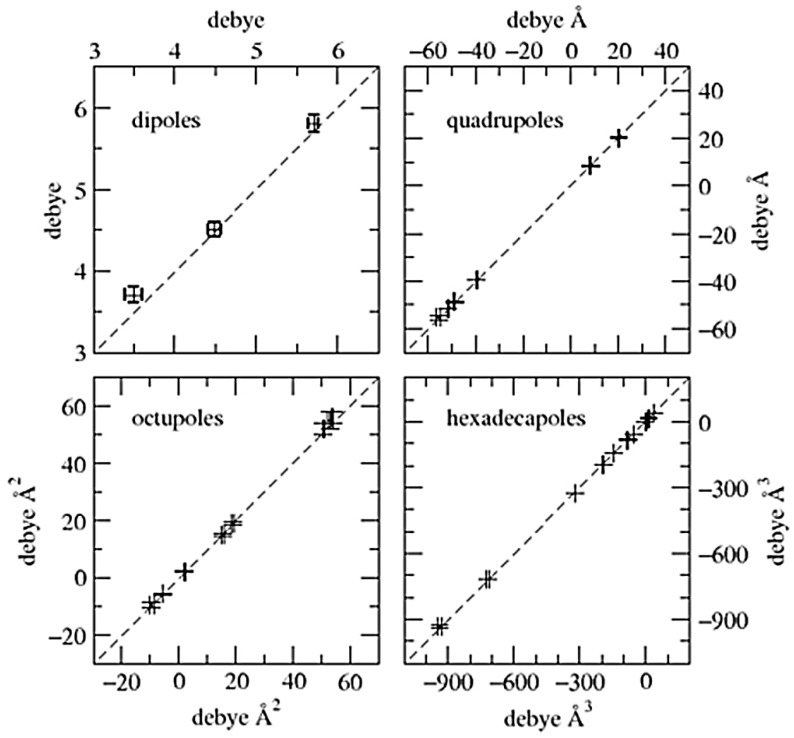
Electrostatic unabridged moments of the 1-MUR molecule removed from the crystal: comparison of values obtained from the unabridged Cartesian moments of Stewart’s atoms (*y* axis) with those obtained from the unabridged Cartesian moments of QTAIM atoms (*x* axis). Data points are represented by their error bars. The dashed 45° degree lines represent perfect agreement. Numerical entries are stored in Table S13 Supplementary Materials.

When molecular boundaries are moved artificially to infinity (by the same criterion as used in the previous section to evaluate the volume of a molecule removed from the crystal), QTAIM molecular moments are identical to Stewart molecular moments within numerical accuracy (Figure 6 and Table S13 Supplementary Materials). A key conclusion is that different charge density partitioning criteria are fully equivalent, as they converge toward identical electrostatic moments of the whole molecule. For example, the Stewart dipole reads 5.8 ± 0.1 D, which is essentially identical to the QTAIM one (5.71 ± 0.6 D, Table S13 Supplementary Materials). Thus, Stewart’s atoms and molecular electrostatic moments derived therefrom describe a molecule rigidly extracted from the crystal. When molecular boundaries are defined by the interatomic surfaces in the crystal, then molecular moments equivalent to the ones measured in the crystal are obtained. As shown in Table S12 Supplementary Materials, the experimental in-crystal QTAIM value is 5.5 ± 0.1 D and differs by only 0.21 D from the QTAIM value for the molecule removed from the crystal (5.71 ± 0.6 D). The difference is of the order of one standard deviation. At variance with other cases [69,70], it can be concluded that the distribution of atomic charges in the 1-MUR molecule is not affected significantly by the crystal environment. 

### 3.9. The Electrostatic Potential Φ_mol_

Maps of the 1-MUR electrostatic potential Φ (**r**), plotted in units of eÅ^−1^, were calculated with VALRAY for the 1-methyluracil molecule extracted from the crystal, (which is taking into account only the contributions of the pseudoatoms of one molecule), after the refinement of each of the four models. 

**Figure 7:**
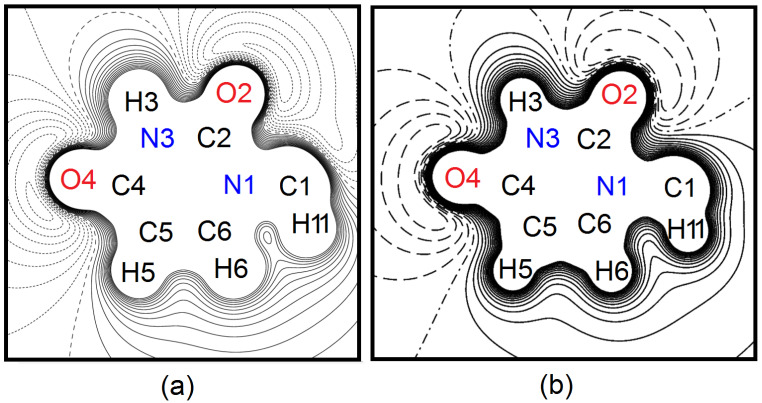
(**a**) Contour map (9 × 9 Å) of the electrostatic potential Φ_mol_ in the molecular plane of model A, i.e., seen along the *c* axis with respect to the crystallographic reference frame. The only atom that is not labelled is H12, which lies out of the (*a*,*b*) plane and is exactly superimposed with its mirror image in the plot. Φ_mol_ is drawn from −0.16 to +0.28 eÅ^−1^ (1 eÅ^−1^ = 332.1 kcal mol^−1^) at intervals of 0.02 eÅ^−1^. Solid lines: positive contours; dotted lines: negative contours; dashed line: Φ_mol_ = 0 eÅ^−1^. (**b**) Same as (**a**), from a periodic CRYSTAL14 calculation on the molecule extracted from the crystal. Dashed lines: negative contours; dash-dotted line: Φ_mol_ = 0 eÅ^−1^.

The four maps are equivalent, with the same features of the one shown in Figure 7a, referring to model A. For the sake of comparison, the same map was computed by a DFT PBE0 simulation using the CRYSTAL14 [71] program and exploiting the Peintinger basis set [72] for the molecule extracted from the crystal (Figure 7b). The minimum in the experimental potential map (Figure 7a) amounts to −219 (38) kJ mol^−1^, about 1.232Å away from O4, which is the oxygen atom involved in the hydrogen bond with the N3–H3 group of the molecule in the crystal related by the inversion center. The values of the minima on the other three maps are −230 (38), −209 (41), and −206 (45) kJ mol^−1^ for models B, C, and D, respectively, and their position is the same, within 0.007Å, as that in the map of the figure. These hydrogen-bonded contacts are crucial to the crystal packing [73,74] because they support 1-MUR pairs in the same plane facing each other, and they underpin the mirror symmetry in the (*a*,*b*) plane. The theoretical map in Figure 7b shows a very good qualitative agreement with the experimental one. The most significant deviations are localized in the region of the C1 methyl group, which bears the largest positive Fourier residual (see above). This confirms that the multipole model, despite the possibility of unmodeled disorder, is accurate enough to catch all the electrostatic features underlying molecular self-recognition in this system. 

## 4. Conclusions

The results of the Hamilton test strongly support the inclusion of anharmonicity into the thermal motion modelling of the 1-MUR crystal, even if the data resolution is not optimal and the dataset suffers from unmodelled disorder. The highly significant improvement of the fit to the observed XRD data occurs at the expense of a large increase of the esds of the refined parameters. It is reasonable to expect a large reduction of these uncertainties with fitting higher resolution data. Upon insertion of the GC cumulants, the residual Δ*ρ* is significantly reduced (by about 30%), but the peak near the CH_3_ group remains unusually high. A proper treatment of disorder in this 1-MUR crystal form is impossible based on the currently available X-ray diffraction data. Rather, dealing with the disorder would require accurate data collection with a modern detector to look for elusive diffuse scattering signatures and/or very weak superlattice reflections [75]. Based on the MD simulation, the disorder in these crystals is likely dynamical and long-range in nature. In any case, no changes in electrostatic properties are apparent upon inclusion of anharmonicity or changes in the multipole model. Different partitioning schemes of EDD all produce consistent molecular electrostatic moments. The distribution of atomic charges in 1-MUR is not affected by the crystal environment in a significant way. The general uniformity of results from different multipole models of increasing complexity, as well as from quantum mechanical calculations, argues in favor of the reliability of the experimentally derived 1-MUR topological and electrostatic parameters.

## Figures and Tables

**Table 1:** Crystal data and details of data collection for 1-methyluracil. Estimated standard deviations in parentheses.

Sample Information	
Empirical formula	C_5_H_6_N_2_O_2_
Formula wt/g mol^−1^	126.12
Crystal system	Orthorhombic
Space group	Ibam
Z	8
Crystal dimensions/mm	0.20 × 0.28 × 0.34
T/K	23 (1)
*a*/Å	13.208 (1)
*b*/Å	13.164 (1)
*c*/Å	6.1620 (6)
V/Å^3^	1071.4 (2)
Dx/g cm^−3^	1.564
F (000)	528
Absorption coeff., µ/mm^−1^	0.124
Data Collection	
Cryostat	He/closed-cycle
Diffractometer	Four-circle
λ/Å	0.71073
Monochromator	Graphite
(sinθ/λ)_max_ (Å^−1^)	1.143
Scan technique	ω/2θ
Scan rate (2θ)/°min^−1^	3
Scan range (2θ)/°	2.5 + S_α1–α2_^†^
No. collected refln.s	11,985
no. unique refln.s	3568
No. obsd refln.s (I > 0, N_obs_)	3344
No. refln.s with I > 3σ (I), N_1_	2614

^†^ S_α1-α2_ is the MoKα_1_-α_2_ separation in the intensity profile.

**Table 2:** Results of the multipolar refinement of the 1-methyluracil structure with VALRAY2000.

Model *^a^*	A C,N,O: *l*_max_ = 3 H: *l*_max_ = 2	B C,N,O: *l*_max_ = 4 H: *l*_max_ = 2	C Model B + *C_ijk_* (All Atoms)	D Model C + *D_ijkl_* (C, N, O)
Scale factor	1.00027 (273)	1.00017 (275)	0.99982 (293)	0.99975 (359)
Extinc. coeff. g_11_	0.527 (9)	0.533 (9)	0.531 (9)	0.529 (9)
No. variables	179	224	312	393
On all 3344 observed (I > 0) data				
ΣwΔ^2^	3062.70858	2920.61101	2780.51474	2645.12507
G.o.f.	0.9837	0.9675	0.9576	0.9468
R (F)	0.0289	0.0285	0.0282	0.0277
R (F^2^)	0.0205	0.0195	0.0191	0.0185
wR (F^2^)	0.0337	0.0329	0.0321	0.0314
On 115 data with (sinθ/λ) ≤ 0.35 (low-angle data)				
R (F)	0.0053	0.0049	0.0047	0.0043
R (F^2^)	0.0120	0.0104	0.0104	0.0100
wR (F^2^)	0.0118	0.0110	0.0104	0.0100
On 663 data with (sinθ/λ) ≤ 0.65 (i.e., within the Cu sphere)				
R (F)	0.0111	0.0103	0.0098	0.0096
R (F^2^)	0.0123	0.0110	0.0106	0.0103
wR (F^2^)	0.0189	0.0171	0.0164	0.0159
On 2681 data with 0.65 < (sinθ/λ) ≤ 1.15 (high-angle data)				
R (F)	0.0389	0.0387	0.0385	0.0379
R (F2)	0.0339	0.0335	0.0330	0.0321
wR (F^2^)	0.0469	0.0465	0.0456	0.0445
On 2614 data with I > 3σ (I)				
R (F)	0.0181	0.0177	0.0173	0.0169
R (F^2^)	0.0188	0.0179	0.0174	0.0168
wR (F^2^)	0.0309	00301	0.0293	0.0285
ΣwΔ^2^	2570.44048	2432.65591	2302.79838	2187.30982
Δρ min	−0.23	−0.22	−0.21	−0.19
at X,Y (e/Å^3^)	0.448; 0.067	0.448; 0.067	0.448; 0.064	0.329; 0.310
Δρ max	+0.53	+0.49	+0.44	+0.37
at X,Y (e/Å^3^)	0.441; 0.097	0.441; 0.097	0.443; 0.095	0.440; 0.095

*^a^* For each model, the maximum order of multipole and Gram–Charlier coefficients is specified for each atomic species (see text).

**Table 3:** Results of the Hamilton significance test [31] for 1-MUR at T = 23 K.

Compared Models		A vs. B	B vs. C	C vs. D
b = dimension of the hypothesis		224 − 179 = 45	312 − 224 = 88	393 − 312 = 81
n-m = degrees of freedom		3344 − 224 = 3120	3344 − 312 = 3032	3344 − 393 = 2951
Actual R-factor ratio ℛ		√ (3062.709/2920.611) = 1.0240	√ (2920.611/2780.515) = 1.0249	√ (2780.515/2645.125) = 1.0253
Significance ℛ_b,n-m,α_	α = 0.001	1.0128	1.0222	1.0213
	α = 0.0005	1.0133	1.0227	1.0219

**Table 4:** QTAIM-estimated atomic charges q (*e*) and volumes V (Å^3^) for the molecule in crystal.

Model	A		B		C		D		Arithmetic Averages	
Atom	q	V	q	V	q	V	q	V	q	V
C1	−0.040	12.03	−0.022	11.88	−0.012	11.58	−0.018	11.54	−0.02 (1)	11.8 (2)
C2	1.369	4.96	1.393	4.89	1.437	4.87	1.390	4.90	1.40 (3)	4.90 (3)
C4	1.125	6.42	1.173	6.38	1.212	6.36	1.232	6.31	1.19 (4)	6.37 (4)
C5	−0.027	12.40	−0.047	12.60	−0.022	12.13	−0.059	12.66	−0.04 (2)	12.5 (2)
C6	0.148	9.69	0.145	9.64	0.146	9.70	0.174	9.63	0.15 (1)	9.66 (3)
N1	−0.962	10.98	−0.952	10.84	−0.978	10.88	−1.003	10.98	−0.97 (2)	10.92 (6)
N3	−1.036	13.69	−1.022	13.36	−1.021	13.29	−1.058	13.41	−1.03 (2)	13.4 (2)
O2	−0.948	16.73	−0.982	16.88	−1.033	16.98	−0.975	16.89	−0.99 (3)	16.87 (9)
O4	−1.005	16.73	−1.053	17.07	−1.071	17.06	−1.045	16.96	−1.04 (2)	17.0 (1)
H3	0.565	1.88	0.563	1.88	0.537	2.00	0.546	2.01	0.55 (1)	1.94 (6)
H5	0.136	6.19	0.128	6.23	0.129	6.19	0.140	6.25	0.133 (5)	6.22 (3)
H6	0.180	5.04	0.183	5.00	0.183	4.95	0.196	4.90	0.185 (6)	4.97 (5)
H11	0.164	4.95	0.169	5.01	0.175	4.87	0.173	4.90	0.170 (4)	4.93 (5)
H12	0.165	6.26	0.161	6.30	0.160	6.34	0.154	6.43	0.160 (4)	6.33 (6)
H12	0.165	6.26	0.161	6.30	0.160	6.34	0.154	6.43	0.160 (4)	6.33 (6)
∑	0	134.21	0	134.26	0	133.53	0	134.16	0	134.0 (3)

**Table 5:** Electrostatic traceless moments of the 1-MUR molecule removed from the crystal. Comparison of PAMoC results from the Stewart DMA of the EDD. Origin: center of mass; system of coordinates: principal axes of inertia of the molecule.

	Model					
<*O* (r)> ^#^	A	B	C	D	Weighted Mean ^‡^	Mulliken DMA ^§^
<q>	0	0	0	0	0	0.00
<x>	3.5 (3)	3.7 (3)	3.8 (4)	3.6 (4)	3.7 (2)	3.35
<y>	4.5 (2)	4.7 (2)	4.4 (2)	4.4 (2)	4.5 (1)	3.58
|**µ**| *	5.7 (3)	6.0 (3)	5.9 (4)	5.7 (4)	5.8 (2)	4.90
<xx>	−9.8 (8)	−9.9 (8)	−9 (1)	−10 (1)	−9.6 (4)	−11.27
<xy>	12.3 (7)	12.8 (7)	12.5 (8)	12.5 (9)	12.5 (4)	9.46
<yy>	13.9 (7)	13.9 (8)	13.3 (9)	13.4 (9)	13.7 (4)	8.60
<zz>	−4.2 (5)	−4.0 (5)	−4.6 (6)	−3.8 (6)	−4.1 (3)	2.67
*α*	6.4 (1)	6.4 (1)	6.3 (1)	6.4 (1)	6.38 (6)	4.91
<xxx>	70 (3)	70 (3)	64 (3)	61 (4)	67 (2)	69.46
<yyy>	−5 (2)	−5 (2)	−5 (2)	−5 (2)	−5 (1)	−10.03
<xyy>	−50 (2)	−51 (2)	−46 (2)	−44 (2)	−48 (1)	−51.30
<xxy>	33 (2)	34 (2)	33 (2)	33 (2)	33 (1)	32.84
<xzz>	−20 (2)	−20 (2)	−18 (2)	−17 (2)	−18.8 (9)	−16.80
<yzz>	−28 (1)	−29 (1)	−28 (1)	−28 (1)	−28.5 (6)	−22.81
<xxxx>	−112 (8)	−126 (8)	−115 (9)	−126 (10)	−119 (4)	−102.94
<yyyy>	31 (6)	22 (6)	23 (7)	18 (7)	24 (3)	18.58
<zzzz>	11 (6)	6 (6)	12 (7)	8 (7)	9 (3)	−18.70
<xxxy>	−40 (7)	−41 (7)	−31 (8)	−30 (8)	−36 (4)	−51.71
<yyyx>	72 (6)	75 (6)	65 (7)	64 (7)	70 (3)	74.62
<xxyy>	45 (7)	55 (7)	52 (8)	58 (8)	52 (4)	32.83
<xxzz>	66 (5)	71 (6)	63 (6)	68 (6)	67 (3)	70.11
<yyzz>	−77 (4)	−77 (4)	−76 (4)	−76 (4)	−77 (2)	−51.41
<zzxy>	−33 (3)	−34 (3)	−34 (4)	−34 (4)	−33 (2)	−22.91

^#^ Expectation value of the moment operator *O* (**r**), which is 1 for charge, **r** for dipole moment, **r** × **r** for quadrupole moment, etc. Units are electrons for charge (*l* = 0), debye for dipole moment (*l* = 1), DebyeÅ or Buckingham for quadrupole moment (*l* = 2), DebyeÅ*^l^* for higher order moments (*l* ≥ 3). ^‡^ The weighted mean is defined as $\bar{a}=\frac{\sum_{i}w_{i}a_{i}}{\sum_{i}w_{i}}$, where $w_{i}=1/\sigma^{2}\left(a_{i}\right)$ and $\sigma \left(a_{i}\right)$ are the weight and the standard deviation of *a_i_*, respectively. The standard error of the weighted mean is $\sigma \left(\bar{a}\right)={\left(\sum_{i}w_{i}\right)}^{-1/2}$. ^§^ Computed from the B3LYP/6-311G (d,p) wave function of the isolated molecule at the crystal geometry obtained with the ORCA program [61]. * Magnitude of the dipole moment: $\left|\mu \right|=\sqrt{\left\langle x^{2}\rangle +\langle y^{2}\rangle +\langle z^{2}\right\rangle }$. ^¶^ Anisotropy of the quadrupole moment tensor **Q**: $a=\sqrt{3Tr\left[Q^{2}\right]-Tr{\left[Q\right]}^{2}/2}$, where Tr[Q]=0 since **Q** is traceless.

## Data Availability

Diffraction data were deposited as Supplementary Materials for this paper.

## Supplementary Materials

The following are available online. Behavior of cell parameters as a function of T. Further tests on the residual density analysis. Details of Molecular Dynamics calculations. Coordinates and thermal motion data. Multipole analysis and electrostatic moments. Comments on the electroneutrality constraints. QTAIM results. Stewart’s unabridged Cartesian moment tensors.

## Sample Availability

Samples of the compounds are not available from the authors.
