# Supplementary material for: Anharmonic Thermal Motion Modelling in the Experimental XRD Charge Density Determination of 1-Methyluracil at T = 23 K
Source: Molecules. 2021 May 21;26(11):3075. doi: 10.3390/molecules26113075 (PMC8196607; doi:10.3390/molecules26113075)
Supplement: Supplementary file 1 [file molecules-26-03075-s001.zip › Destro_Roversi_Barzaghi_Lo-Presti_Supporting_Information.pdf]

Anharmonic thermal motion modelling in the experimental XRD charge density determination of 1-Methyluracil at T=23 K

Riccardo Destro,[a,*] Pietro Roversi,[b] Mario Barzaghi[c] and Leonardo Lo Presti[a,d]

[a] Chemistry Department, Università degli Studi di Milano, Via Golgi 19, 20133 Milano, Italy
[b] Leicester Institute of Structural and Chemical Biology, Department of Molecular and Cell Biology, University of Leicester, Lancaster Road, Leicester LE1 7HB, UK
[c] Consiglio Nazionale delle Ricerche (CNR), Piazzale Aldo Moro 7, 00185 Roma, Italy
[d] Istituto Nazionale di Fisica Nucleare (INFN), Laboratori Nazionali di Frascati, Frascati (Roma)

Author E-mail addresses:
Riccardo Destro: riccardo.destro@unimi.it
Pietro Roversi: pr159@leicester.ac.uk
Mario Barzaghi: mario.barzaghi@cnr.it
Leonardo Lo Presti: leonardo.lopresti@unimi.it

* Author to whom correspondence should be addressed.

SUPPORTING INFORMATION

INDEX

S1. Cell parameters 3

S2. Residual density analysis 4

S3. Solid state Molecular Dynamics 8

S4. Structural and thermal motion results 13

S5. Multipole analyis and electrostatic moments 16

S5.1 The electroneutrality constraint. 16

S5.2 QTAIM results 18

S5.3 Stewart's unabridged Cartesian moment tensors 22

S1. Cell parameters

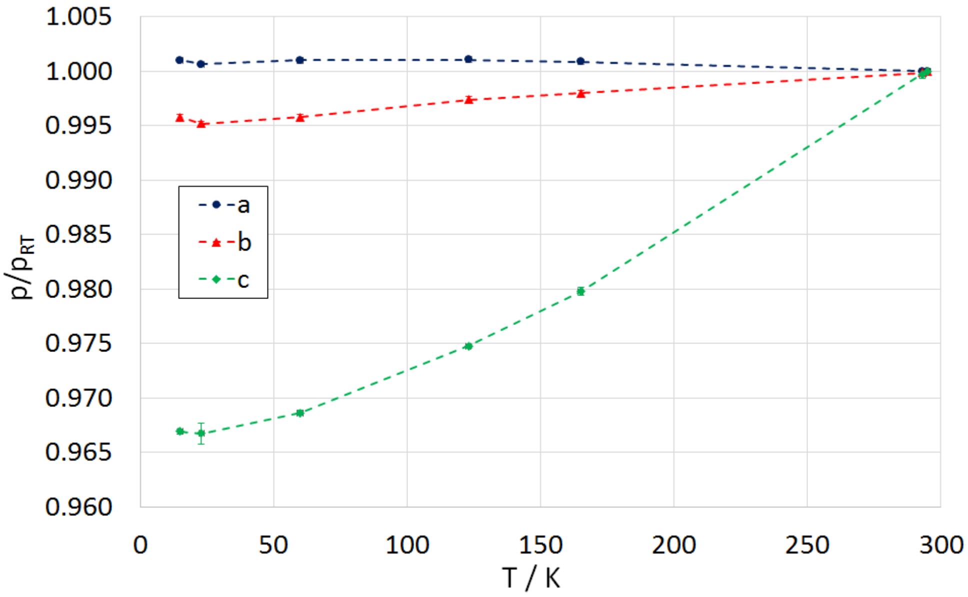

Figure S1. Relative changes of the cell edges of the N–methyluracil crystal as a function of T with respect to room temperature (RT). Blue dots: *a*. Red triangles: *b*. Green diamonds: *c*. Dashed lines serve only as a guide for the eye. The estimated standard deviations are in most cases of the same order of magnitude as the dimension of the dot.

Table S1. Cell edges of N–Methyluracil as a function of T.

T (K)	*a*	*b*	*c*	*V*	Ref[a]
295	13.200(2)	13.228(2)	6.374(1)	1113.0(6)	Av. Mi-Pa
293	13.200(3)	13.226(2)	6.372(2)	1112.4(8)	MC
165	13.211(3)	13.201(3)	6.245(2)	1089.2(8)	Mi
123	13.214(3)	13.193(3)	6.213(1)	1083.1(7)	MC
60	13.213(3)	13.172(3)	6.174(1)	1074.5(7)	MC
23	13.208(1)	13.164(1)	6.1620(6)	1071.4(2)	This work
15	13.213(2)	13.172(2)	6.163(1)	1072.6(6)	MC

[a] MC McMullan, R.K.; Craven, B.M. Crystal structure of 1-methyluracil from neutron diffraction at 15, 60 and 123 K. *Acta Crystallogr. Sect. B Struct. Sci.* **1989**, *45*, 270–276
Av. Mi - Pa = average estimate from several cells measured by single crystal X-ray diffraction at Caltech (Pasadena) and Unimi (Milano).

S2. Residual density analysis

Table S2 shows the relevant RDA parameters for the $\Delta\rho$ distribution of each model, as computed from a 151^3 grid points of residual density over the whole unit cell volume.

Table S2. Residual density analysis (RDA) parameters in the whole unit cell, based on 151 x 151 x 151 grid points of $(F^o - F^c)$–based $\Delta\rho$ for each of the three crystallographic directions.

Model →	A	B	C	D
e_{net}/e [a]	$4 \cdot 10^{-4}$	$-5 \cdot 10^{-3}$	$-3 \cdot 10^{-3}$	$-1 \cdot 10^{-2}$
e_{gross}/e [b]	25.8639	25.6650	25.5650	25.2104
$d^f(0)$ [c]	2.6515	2.6528	2.6526	2.6527

[a] Net residual electrons, equation (1)
[b] Total residual electrons, equation (2)
[c] Fractal dimension for $\Delta\rho = 0$ (see text)

For a finite collection of grid elements, e_{net}, the amount of net residual electrons, and e_{gross}, the total residual electrons, read[1]:

$$e_{net} = \frac{V}{N}\sum_{k=1}^{N}\Delta\rho(k) \qquad (S1)$$

$$e_{gross} = \frac{V}{2N}\sum_{k=1}^{N}|\Delta\rho(k)| = e_{gross,noise} + e_{gross,model} \qquad (S2)$$

where V is the integrated volume, N the total number of grid points and $\Delta\rho(k)$ the residual electron density corresponding to the k–th finite volume element. As for the present case, e_{net} ranges from 10^{-2} to 10^{-4} electrons over the whole crystallographic unit cell. This can be taken as the maximum accuracy error in $\Delta\rho$ due to numerical discretization. On the other hand, e_{gross} includes the inadequacies due to random noise, plus the systematic ones due to data reduction and model errors (equation 2). This quantity steadily decreases with the complexity of the multipole model, as expected, even though it remains quite high.

A plot of the fractal (or Hausdorff) dimension distribution of the residual density, $d^f(\Delta\rho)$ is shown in Figure S2. This quantity expresses the ability of specific $\Delta\rho$ isosurfaces to cover the whole space, and is maximum for the $\Delta\rho$=0 one.[1] The closer $d^f(0)$ to 3, the closer the $\Delta\rho$=0 region to cover the three–dimensional Euclidean manifold.

[1] Meindl, K.; Henn, J. Foundations of residual-density analysis. *Acta Crystallogr. Sect. A Found. Crystallogr.* **2008**, *64*, 404–418.

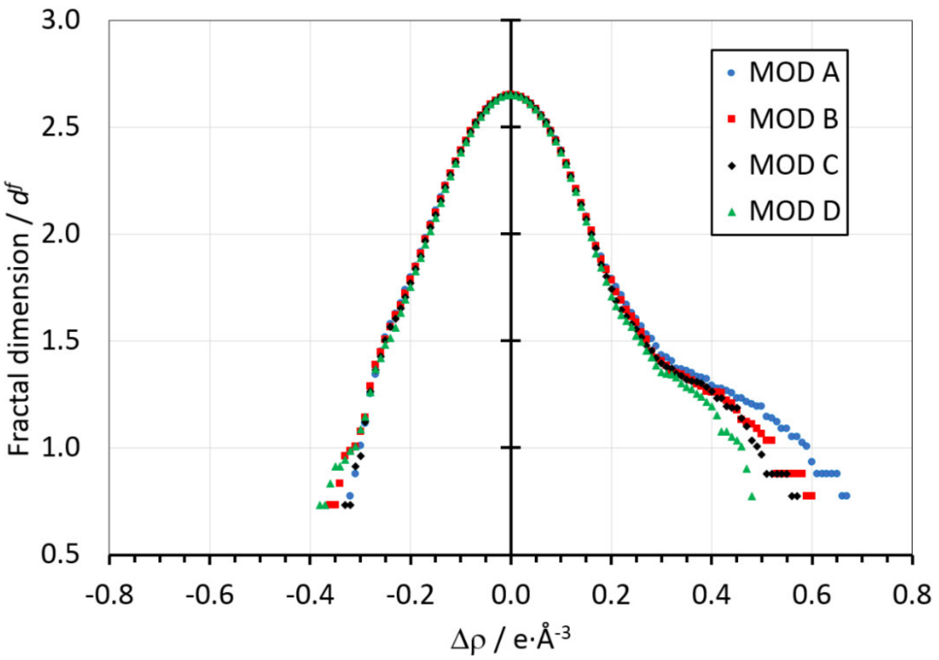

Figure S2. Fractal dimension distributions of the experimental residual charge density in N–methyl uracil at T = 23(1) K, depending on the multipole model employed to fit experimental data. Blue dots: Model A. Red squares: Model B. Black rhombi: Model C. Green triangles: Model D.

Noise and model bias lower the maximum attainable value, which in the present case is invariably close to 2.65 (Table S2). The expected shape for a bias–free $d^f(\Delta\rho)$ plot should be an inverted parabola, the spread over the Δρ axis being proportional to the amount of random noise. This is not the case for the models A–D, (Figure S2), which show a shoulder for Δρ>0 residuals. This is consistent with the large $e_{gross} \approx 25\ e$ above discussed. A flat shoulder typically arises due to inadequacies either in the scale factor, or in the radial parameter refinement.[1] This may indicate that some model shortfalls persist, even though this feature is significantly reduced upon the introduction of higher order cumulants (Models C and D). If only the 2614 data with I > 3 σ(I) are considered (Table 2 in the main text), the Δρ distribution is flatter (Figure S3 SI), as expected. Accordingly, e_{gross} is reduced (Table S3 SI) and the corresponding Hausdorff distribution is narrower (Figure S4 SI). However, the right shoulder persists, demonstrating that it is not connected with noise.

Table S3. Residual density analysis (RDA) parameters in the whole unit cell, based on 151 x 151 x 151 grid points of $(F^o - F^c)$–based $\Delta\rho$ for each of the three crystallographic directions. Only 2614 independent squared structure factor amplitudes with I > 3 σ(I) were considered.

Model →	A	B	C	D
e_{net}/e [a]	$2{\cdot}10^{-2}$	$1{\cdot}10^{-2}$	$2{\cdot}10^{-2}$	$-1{\cdot}10^{-4}$
e_{gross}/e [b]	16.192	15.934	15.706	15.385
$d^f(0)$ [c]	2.6494	2.6509	2.6504	2.6507

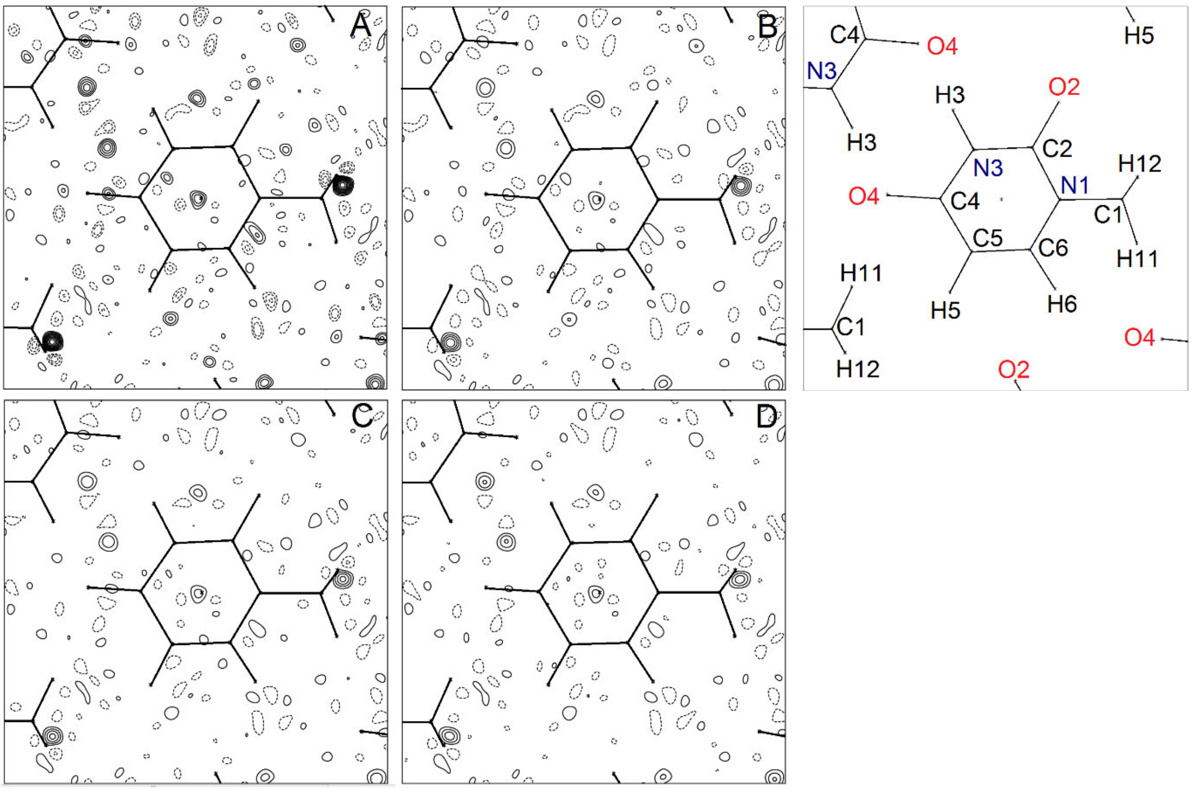

Figure S3. Same as Figure 4 (main text), computed from 2614 independent squared structure factor amplitudes with I > 3 σ(I).

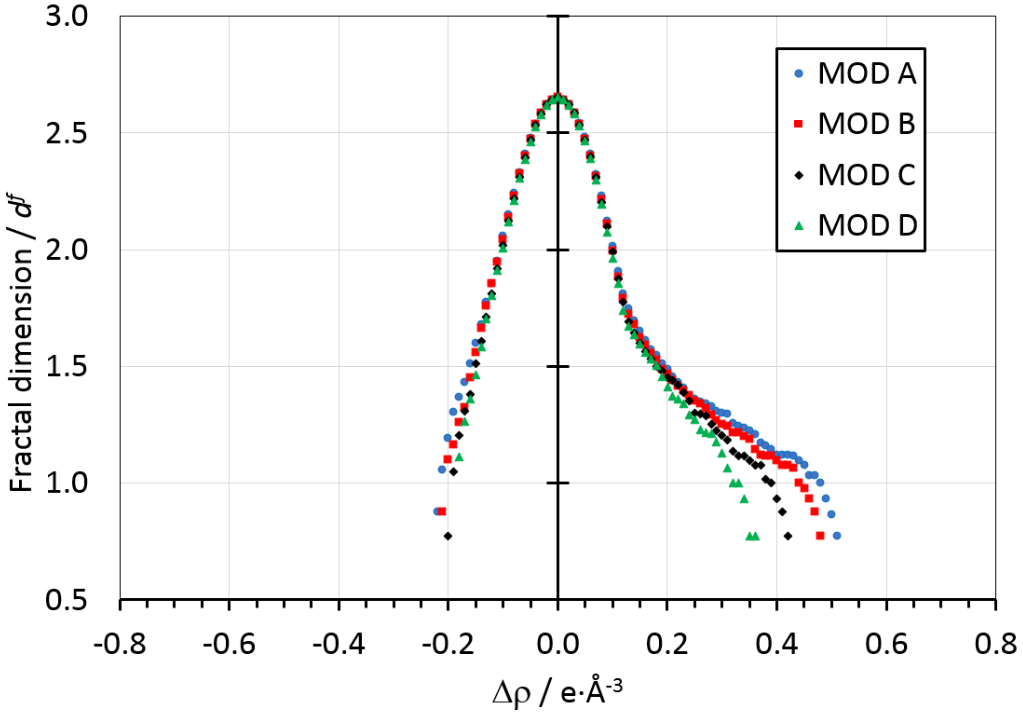

Figure S4. Fractal dimension distributions of the experimental residual charge density in N–methyl uracil at T = 23(1) K, depending on the multipole model employed to fit experimental data. Only 2614 independent squared structure factor amplitudes with I > 3 σ(I) were considered. Blue dots: Model A. Red squares: Model B. Black rhombi: Model C. Green triangles: Model D.

To estimate the relative importance of noise and bias contributions (eq. S2) to the total residual density we computed a full set of predicted structure factors, F^c, from the best model D. Then, we repeated the RDA analysis as a function of the amount of random gaussian noise added to the synthetic data (see ref. 1 for full details of the procedure). Results are shown in Table S4. Simulated noise affects the same way the two models. For noise–free (p_1=0.00) simulated data, e_{gross} attains its minimum value but is still more than 2 electrons high. This implies that 0.5 % of electrons at least are intrinsically misplaced by our models, even though the overall Δρ map is featureless. Moreover, the Hausdorff dimension stands around 2.60 or 2.61 when p_1>0, which is lower than the experimental estimates (~2.65). All these evidences point out that Models A–D are slightly overfitted, that is, a part of the noise is absorbed into the density model. Figure S5 shows the residual density features in the (*a*, *b*) plane for the p_1=0.05 noise level, which corresponds to the experimental flatness (Table S4).

Table S4. Residual density descriptors applied to simulated noisy data for N–methyluracil (Models A and D) as a function of a noise control parameter p_1 [46]. The higher p_1, the noisier the data. The residual density in the whole unit cell was computed from a grid of 151 x 151 x 151 grid points. Ten cycles of least–squares refinement with VALRAY were applied after the addition of noise.

p_1	Model A				Model D			
	e_{gross}/e	$\%e_{gross}$[a]	$d^f(0)$	Δρ / e[b]	e_{gross}/e	$\%e_{gross}$[a]	$d^f(0)$	Δρ / e[b]
0.000	2.45	0.5	-[c]	0.03	2.15	0.4	-[c]	0.03
0.010	8.55	1.6	2.5941	0.17	7.64	1.4	2.6121	0.16
0.025	19.69	3.2	2.6101	0.45	20.67	3.9	2.5996	0.45
0.050	43.73	8.3	2.6042	0.95	42.77	8.1	2.5801	0.99
0.075	62.07	11.8	2.6076	1.23	58.88	11.2	2.6047	1.41
Experimental	25.86	4.9	2.6515	0.99	25.21	4.8	2.6527	0.87

[a] Percent of e_{gross} with respect to the total number of electrons in cell, 528.
[b] Flatness of the residual density, defined as $\Delta\rho_{max} - \Delta\rho_{min}$.
[c] Too few points in the distribution for meaningful statistics.

All peaks range between ±0.5 e/Å^3 and occupy random positions in the plane of the picture: this means that the structured residual noted in Figure 4 above have nothing to do with noise and, rather, they must have a true physical origin. It is also important to stress that the overfitting is not due to the addition of higher order cumulants, as the much simpler Model A equally suffers the same problem.

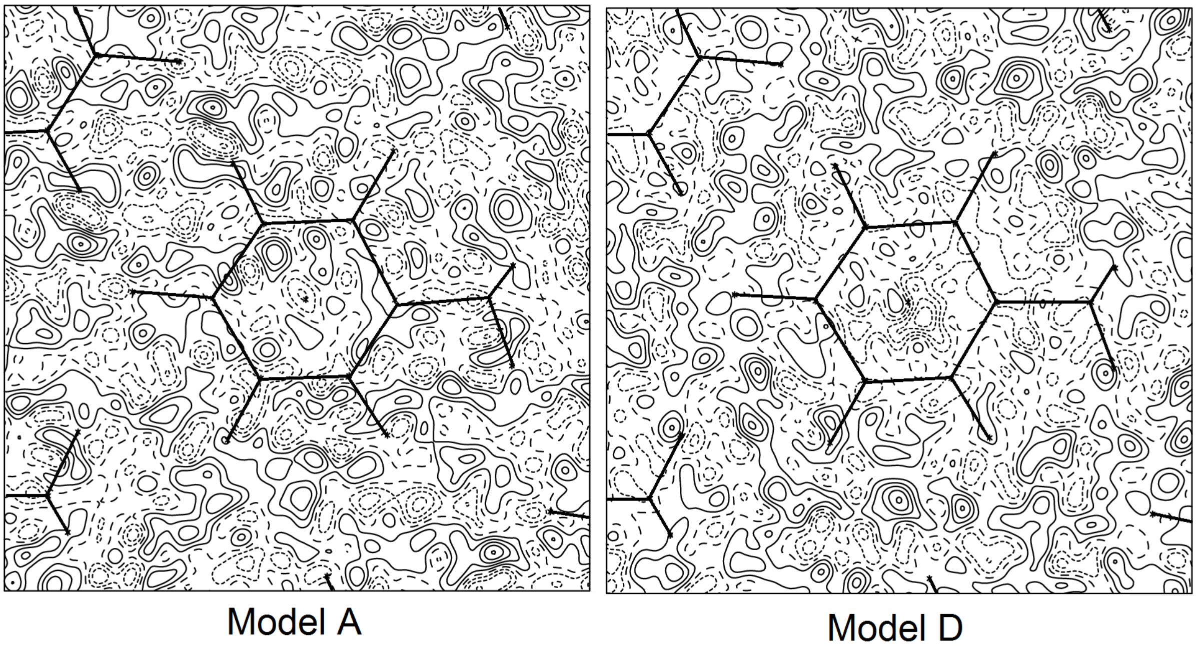

Figure S5. Residual density map in the main molecular plane for Models A and D applied on sets of computed structure factor amplitudes, to which a random gaussian noise according to Meindl & Henn was added (see main text). Atom numbering is the same shown in Figure 3. The noise control parameter is $p_1 = 0.05$. The map is 9 x 9 Å wide and is plotted down the *c* axis. The origin is at the centre of coordinates of the 6–membered ring. Contour levels are drawn at steps of 0.1 e·Å^{-3} as full (dashed) lines if positive (negative) and range from –0.5 e·Å^{-3} to +0.5 e·Å^{-3}.

S3. Solid state Molecular Dynamics

To gain insights on possible reasons for the model inadequacies discussed in Section S2, we performed 400 ps–long NpT (isothermal–isobaric ensemble) runs of classical molecular dynamics (MD) on crystalline 1–methyluracil at time steps of 2 fs. The MiCMoS package[2–4] was used throughout, starting from the present crystallographic structure. Simulations were carried out in a 2x2x4 periodic simulation box with 128 molecules and exploited the Lennard–Jones–Coulomb force field.[5] Temperature was set at 23 K using a weak–coupling Berendsen thermostat, while isotropic pressure scaling was ensured through a Parrinello–Rahman algorithm.[4] The simulations reproduced the experimntal lattice parameters and the crystal density with a reasonable degree of accuracy, with deviations not larger than 5–6 % on the cell

[2] Gavezzotti, A.; Lo Presti, L. Molecular dynamics simulation of organic crystals: introducing the CLP-dyncry environment. *J. Appl. Crystallogr.* **2019**, *52*, 1253–1263

[3] Lo Presti, L.; Gavezzotti, A. MiCMoS - Milano Chemistry Molecular Simulation. MiCMoS user's manual v1.2, 2020. See https://sites.unimi.it/xtal_chem_group 2021

[4] Rizzato, S.; Gavezzotti, A.; Lo Presti, L. Molecular Dynamics Simulation of Molecular Crystals under Anisotropic Compression: Bulk and Directional Effects in Anthracene and Paracetamol. *Cryst. Growth Des.* **2020**, *20*, 7421–7428

[5] Gavezzotti, A.; Lo Presti, L.; Rizzato, S. Mining the Cambridge Database for theoretical chemistry. Mi-LJC: a new set of Lennard-Jones–Coulomb atom–atom potentials for the computer simulation of organic condensed matter. *CrystEngComm* **2020**, *22*, 7350–7360

edges and ± 0.5 deg on the cell angles (see Table S5 SI). After ~30 ps, the system was fully equilibrated (Figure S6). Tables S6 and S7 report the input and topology files we employed to generate the trajectories.

Table S5. Experimental *vs.* classical Molecular Dynamics (MD)–predicted lattice parameters. Estimated standard deviations of MD values refer to the time average over the last 300 ps of the trajectory, when the system is fully equilibrated. See text for the details of the calculation.

	a / Å	b / Å	c / Å	α / deg	β / deg	γ / deg	ρ / g·cm^{-3}
Experimental	13.208(1)	13.164(1)	6.1620(6)	90.0	90.0	90.0	1.564
MD	12.612(13)	13.423(2)	6.505(5)	90.49(3)	89.83(2)	89.91(12)	1.522(1)
Δ %	–4.5	+2.0	+5.6	+0.5	–0.2	–0.1	–2.7

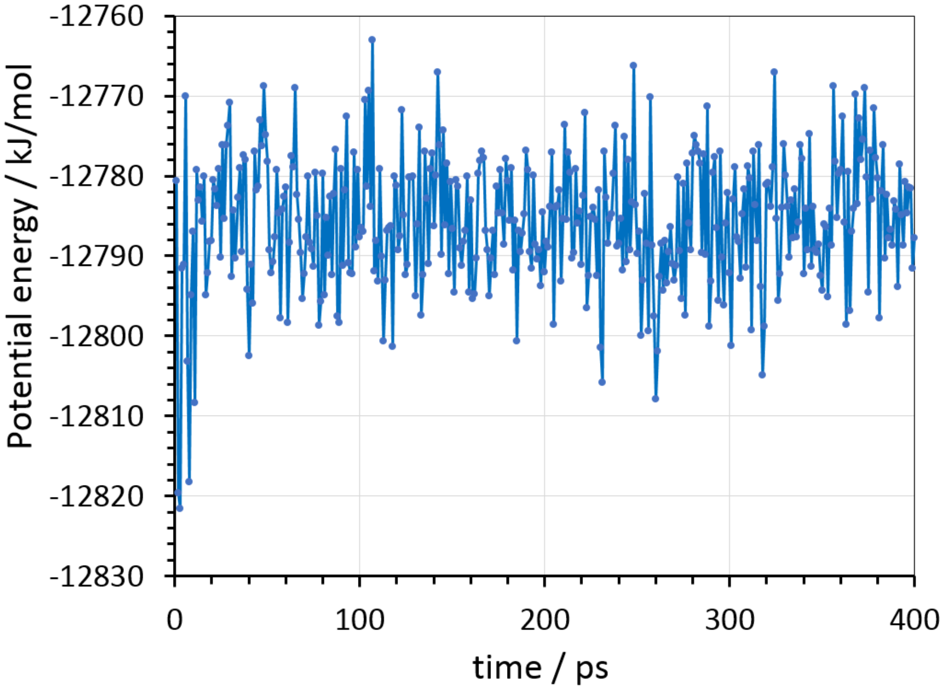

Figure S6. Potential energy (kJ/mol) *vs.* t (ps). The system is fully equilibrated after ~ 40 ps.

Table S6. Input for Molecular Dynamics (.mdi file, see MiCMoS manual at https://sites.unimi.it/xtal_chem_group/index.php/research/5-micmos)

```
MUR 23 K LJC PR no stress LF weakC
# n.steps  irvel  ipri  ibox idstr  timestep   Emolim  LF/VV
  200000     0      0     1    0    0.002      0.0      0
# cutoffu cutoffv cutoffuv factin  ipots
   12.0    0.0     0.0      0.7      1
#   N(T)    Tset Tstart Trelax  0/1/2weak/stiff/CSVR
    100     23    23     0.6      0
#   N(P)    Pset  comprs  0/1ianis ipr   ww    iextstr + stra11 22, 33, 12 13 23, GPa
     50     1.0   0.0       1       1    3.0     0
#   N(com)  nwbox   nwre   npri
     50     1000    1000   500
#   Nrot-ev romin   maxs   stepr   Rev    fact   icut
    200     -15     11     3.0    30.0    0.9    2
```

Table S7. Topology for Molecular Dynamics

Atomic charges come from a fit against the electrostatic potential of the isolated molecule computed at the perturbative MP2 6-31G* level (see MiCMoS Manual, https://sites.unimi.it/xtal_chem_group/index.php/research/5-micmos)

```
#METURA23   'I b a m topology
  15
   1   0.00000   0.77592  -2.54454  13   -0.2950
   2   0.00000  -0.79527  -0.70448  10    0.6791
   3   0.00000  -0.00932   1.63170  10    0.7381
   4   0.00000   1.33850   1.12779  12   -0.4709
   5   0.00000   1.54493  -0.21110  12   -0.0202
   6   0.00000   0.51772  -1.11491  17   -0.0532
   7   0.00000  -0.99419   0.65933  21   -0.6635
   8   0.00000  -1.73204  -1.50077  27   -0.5320
   9   0.00000  -0.32808   2.83602  27   -0.5306
  10  -0.88184   0.33888  -2.98932   3    0.1333
  11   0.00000   1.84199  -2.71783   3    0.1293
  12   0.88184   0.33888  -2.98932   3    0.1338
  13   0.00000   2.17625   1.80945   2    0.2047
  14   0.00000   2.56019  -0.57948   2    0.1648
  15   0.00000  -1.95081   0.95058   7    0.3822
   0  nslav-u
   0  ncore-v
   0  nslav-v
 109.4    0.0 volu-u,volu-v
  15  nstr-u
   1    6   1.453  3184.7   C- N
   1   10   1.080  3600.0   C- H
   1   11   1.080  3600.0   C- H
   1   12   1.080  3600.0   C- H
   2    6   1.376  5008.4   C- N
   2    7   1.378  4947.0   C- N
   2    8   1.229  7907.0   C- O
   3    4   1.439  3850.2   C- C
   3    7   1.384  4810.6   C- N
   3    9   1.246  7541.7   C- O
   4    5   1.355  5623.9   C- C
   4   13   1.080  3600.0   C- H
   5    6   1.368  5184.0   C- N
   5   14   1.080  3600.0   C- H
   7   15   1.000  5300.0   N- H
   0  nstr-v
  24  nbend-u
   1    6    2  118.00   590.2   C- N- C
   1    6    5  121.00   618.1   C- N- C
   2    6    5  121.00   618.1   C- N- C
   2    7    3  126.00   664.5   C- N- C
   2    7   15  115.00   460.0   C- N- H
   3    4    5  119.00   576.7   C- C- C
   3    4   13  120.00   505.0   C- C- H
   3    7   15  118.00   460.0   C- N- H
   4    3    7  115.00   562.3   C- C- N
   4    3    9  125.00   763.8   C- C- O
   4    5    6  123.00   636.7   C- C- N
   4    5   14  119.00   517.5   C- C- H
   5    4   13  120.00   505.0   C- C- H
   6    1   10  109.00   642.5   N- C- H
   6    1   11  109.00   642.5   N- C- H
   6    1   12  109.00   642.5   N- C- H
   6    2    7  116.00   571.6   N- C- N
   6    2    8  122.00   702.0   N- C- O
   6    5   14  119.00   517.5   N- C- H
   7    2    8  122.00   702.0   N- C- O
   7    3    9  120.00   702.0   N- C- O
  10    1   11  109.00   470.0   H- C- H
  10    1   12  109.00   470.0   H- C- H
  11    1   12  109.00   470.0   H- C- H
```

```
  0   nbend-v
 13   ntors-u
11    1    6    2    2.00    -1.0     2.0    H- C- N- C N-Me
 7    2    6    1 100.00     1.0     1.0    N- C- N- C
 6    2    7    3 100.00    -1.0     1.0    N- C- N- C
 2    6    7    8 1600.0    -1.0     1.0    C- N- N- O Discourages oop bending of C=O
 7    3    4    5 100.00    -1.0     1.0    N- C- C- C
 4    3    7    2 100.00    -1.0     1.0    C- C- N- C
 3    4    7    9 1600.0    -1.0     1.0    C- C- N- O Discourages oop bending of C=O
 3    4    5    6 100.00    -1.0     1.0    C- C- C- N
 4    3    5   13 1600.0    -1.0     1.0    C- C- C- H
 4    5    6    1 100.00     1.0     1.0    C- C- N- C
 5    4    6   14 1600.0    -1.0     1.0    C- C- N- H
 6    1    2    5 1600.0    -1.0     1.0    N- C- C- C Discourages oop bending of C-Me
 7    2    3   15 1600.0    -1.0     1.0    N- C- C- H
  0   ntors-v
  0   nlist-u
  0   nlist-v
 0.410    235.0    650.0    77000.0
  0   nextr
```

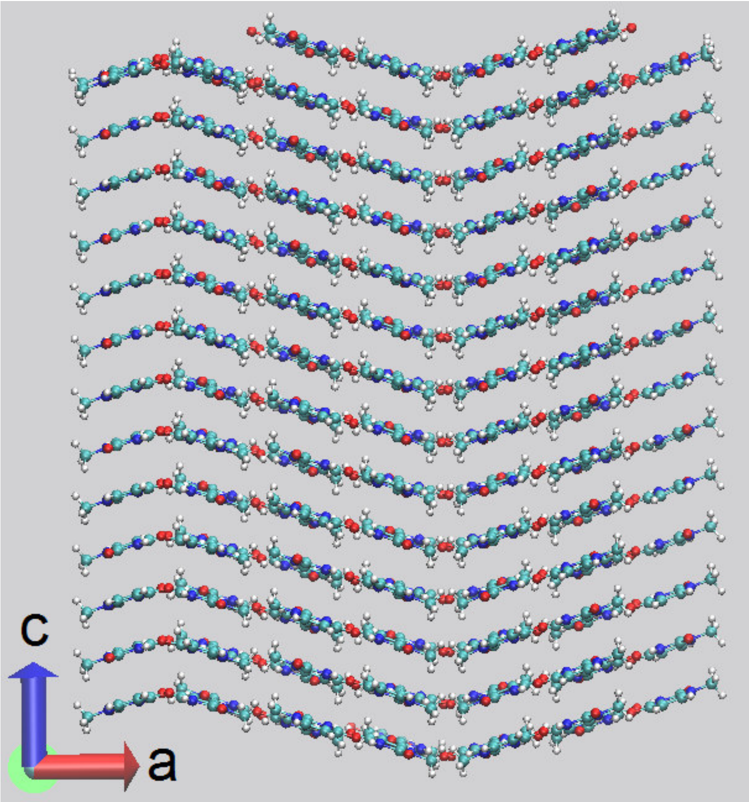

Figure S7. Mean MD simulation box, as obtained from the time average of frames over the last 300 ps of the trajectory. The box contains 3x3x7 crystallographic unit cells and 504 molecules. Apart the box dimensions, the MD simulation was carried out with the same parameters and specs as that discussed in the main text (Figure 6). The long–range superstructure modulation is still present.

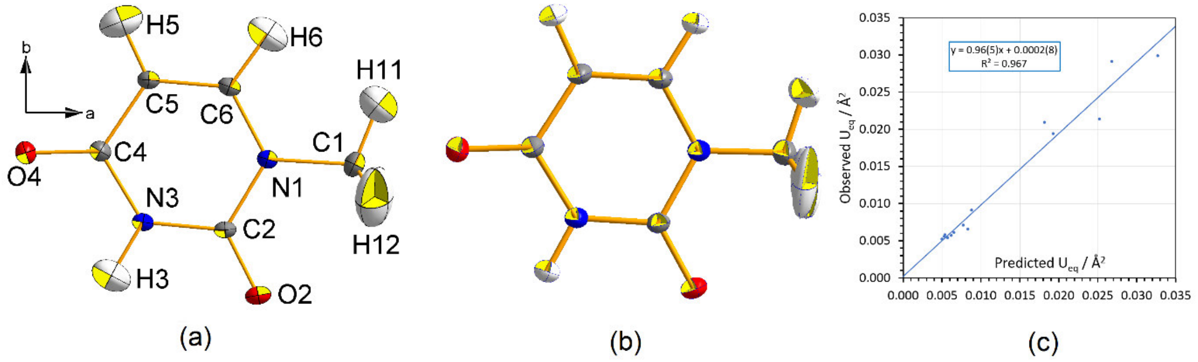

Figure S8. Comparison of experimental thermal ellipsoids (99 % probability level) of N–Methyluracil at 23 K (a) with the MD–predicted ones (b), as computed used the model discussed in the main text (2x2x4 box, 128 molecules). Panel (c) shows the correlation among experimental *vs.* predicted U_{eq} estimates after rescaling of MD quantities to bring all the thermal parameters on a comparable scale. As expected, lighter hydrogen atoms (upper right part of the graph) are more prone to deviations. However, the linear correlation is very good, with slope and intercept equal to 1.0 and 0.0 within 1 standard deviation. MD scale factors were computed by averaging U_{eq}(experimental)/ U_{eq}(MD) ratios over the same class of atoms. They read 1.825 for C, N and O, 1.278 for aromatic H and 5.439 for methyl hydrogens.

S4. Structural and thermal motion results

Table S8. Coordinates (X, Y) and Uij's (×100) from the refinement of models A, B, C and D and from the neutron study at T=15K

Atom	Model	X	Y	U11	U22	U33	U12
C1	A	0.39609(2)	0.12069(2)	0.527(6)	0.716(6)	1.370(9)	-0.002(5)
	B	0.39610(2)	0.12067(2)	0.527(6)	0.714(6)	1.373(8)	-0.005(4)
	C	0.39598(5)	0.12069(5)	0.531(6)	0.712(6)	1.376(9)	-0.006(5)
	D	0.39591(5)	0.12074(5)	0.50(2)	0.68(2)	1.55(4)	0.11(2)
	N	0.39635(6)	0.12063(6)	0.57(3)	0.65(3)	1.32(4)	0.00(3)
C2	A	0.23239(1)	0.03765(1)	0.539(5)	0.345(4)	0.638(6)	0.003(4)
	B	0.23240(1)	0.03765(1)	0.539(5)	0.348(4)	0.636(6)	0.004(4)
	C	0.23245(4)	0.03755(4)	0.537(5)	0.351(5)	0.636(6)	0.004(4)
	D	0.23246(5)	0.03758(5)	0.55(2)	0.30(2)	0.72(2)	0.01(2)
	N	0.23241(5)	0.03763(6)	0.46(3)	0.38(3)	0.62(3)	-0.01(2)
C4	A	0.07446(1)	0.13740(1)	0.490(5)	0.426(5)	0.644(6)	-0.036(4)
	B	0.07445(1)	0.13741(1)	0.491(5)	0.427(5)	0.642(6)	-0.033(4)
	C	0.07449(4)	0.13741(4)	0.494(5)	0.426(5)	0.638(6)	-0.033(4)
	D	0.07457(5)	0.13740(4)	0.50(2)	0.45(2)	0.66(2)	-0.04(2)
	N	0.07445(5)	0.13743(6)	0.43(3)	0.39(3)	0.59(3)	-0.02(2)
C5	A	0.13559(1)	0.22790(1)	0.553(5)	0.406(5)	0.874(7)	-0.004(4)
	B	0.13559(2)	0.22790(1)	0.551(5)	0.404(5)	0.878(7)	-0.004(4)
	C	0.13569(5)	0.22792(4)	0.553(5)	0.405(5)	0.875(7)	-0.004(4)
	D	0.13567(5)	0.22793(4)	0.54(2)	0.35(2)	0.94(3)	-0.01(2)
	N	0.13558(6)	0.22795(6)	0.51(3)	0.44(3)	0.80(3)	0-00(3)
C6	A	0.23788(1)	0.21935(1)	0.531(5)	0.400(5)	0.760(6)	-0.053(4)
	B	0.23787(1)	0.21935(1)	0.531(5)	0.397(5)	0.760(6)	-0.053(4)
	C	0.23796(5)	0.21926(4)	0.529(5)	0.395(5)	0.762(7)	-0.052(4)
	D	0.23794(5)	0.21925(4)	0.56(2)	0.39(2)	0.77(2)	-0.08(2)
	N	0.23792(6)	0.21938(6)	0.48(3)	0.41(3)	0.78(3)	-0.03(2)
N1	A	0.28610(1)	0.12742(1)	0.467(4)	0.394(4)	0.777(6)	-0.040(4)
	B	0.28610(1)	0.12743(1)	0.466(4)	0.392(4)	0.776(6)	-0.036(4)
	C	0.28608(5)	0.12725(4)	0.467(4)	0.391(4)	0.778(6)	-0.036(4)
	D	0.28610(5)	0.12722(4)	0.46(2)	0.37(2)	0.84(3)	-0.03(2)
	N	0.28611(4)	0.12738(4)	0.48(2)	0.42(2)	0.78(2)	-0.03(2)
N3	A	0.12865(1)	0.04740(1)	0.487(4)	0.381(4)	0.833(6)	-0.041(4)
	B	0.12866(1)	0.04740(1)	0.486(4)	0.382(4)	0.830(6)	-0-040(4)
	C	0.12858(5)	0.04732(4)	0.483(4)	0.382(4)	0.831(6)	-0.040(4)
	D	0.12859(5)	0.04731(5)	0.51(2)	0.34(2)	0.90(2)	-0.03(2)
	N	0.12863(4)	0.04747(4)	0.52(2)	0.38(2)	0.84(2)	0.01(2)
	A	0.27432(1)	-0.04560(1)	0.703(5)	0.389(4)	1.058(7)	0.109(4)
	B	0.27432(1)	-0.04560(1)	0.701(5)	0.386(4)	1.060(6)	0.109(4)
O2	C	0.27440(5)	-0.04580(5)	0.702(5)	0.386(4)	1.061(7)	0.113(4)
	D	0.27436(5)	-0.04577(5)	0.74(2)	0.37(2)	1.02(3)	0.07(2)
	N	0.27440(7)	-0.04554(6)	0.67(3)	0.38(3)	1.04(4)	0.09(3)
	A	-0.01945(1)	0.13539(1)	0.454(4)	0.587(4)	0.940(6)	-0.025(4)
	B	-0.01945(1)	0.13538(1)	0.452(4)	0.590(4)	0.939(6)	-0.024(4)
O4	C	-0.01969(5)	0.13538(5)	0.456(4)	0.591(4)	0.940(6)	-0.029(4)
	D	-0.01967(5)	0.13539(5)	0.47(2)	0.57(2)	0.93(3)	-0.04(2)
	N	-0.01942(6)	0.13526(6)	0.38(4)	0.57(3)	0.89(4)	-0.03(3)

Table S9. Bond distances (Å) and relevant intermolecular contacts (Å).

	Model				Arithmetic average	Neutron diffr. at 15 K
	A	B	C	D		
Bond						
C1 - N1	1.4555(3)	1.4557(3)	1.4542(9)	1.4529(9)	1.455(1)	1.459(1)
C2 - N1	1.3782(3)	1.3784(3)	1.3770(8)	1.3763(9)	1.377(1)	1.379(1)
C2 - N3	1.3762(3)	1.3761(3)	1.3779(8)	1.3779(9)	1.377(1)	1.377(1)
C2 - O2	1.2280(3)	1.2278(3)	1.2292(8)	1.2288(9)	1.2284(7)	1.228(1)
C4 - C5	1.4391(3)	1.4391(3)	1.4397(8)	1.4392(8)	1.4393(3)	1.440(1)
C4 - N3	1.3842(3)	1.3844(3)	1.3845(8)	1.3840(9)	1.3843(2)	1.384(1)
C4 - O4	1.2407(3)	1.2406(3)	1.2443(8)	1.2450(9)	1.243(2)	1.241(1)
C5 - C6	1.3557(3)	1.3556(3)	1.3556(9)	1.3557(9)	1.35565(6)	1.357(1)
C6 - N1	1.3676(3)	1.3674(3)	1.3678(8)	1.3683(9)	1.3678(4)	1.369(1)
C1 - H11	1.0824(2)	1.0826(2)	1.0831(7)	1.0827(7)	1.0827(3)	1.083(2)
C1 - H12	1.0890(1)	1.0888(2)	1.0894(4)	1.0900(4)	1.0893(5)	1.088(1)
C5 - H5	1.0812(2)	1.0813(2)	1.0816((6)	1.0814(6)	1.0814(2)	1.081(2)
C6 - H6	1.0878(2)	1.0878(2)	1.0881(6)	1.0884(6)	1.0880(3)	1.088(2)
N3 - H3	1.0413(2)	1.0414(2)	1.0399(6)	1.0398(6)	1.0406(9)	1.043(2)
Hydrogen bond						
N3...O4'	2.8054(3)	2.8054(3)	2.8024(9)	2.8025(9)	2.804(2)	2.806(1)
H3...O4'	1.7642(2)	1.7641(2)	1.7625(6)	1.7627(7)	1.7634(9)	1.764(2)
Intermolecular C-H...O' contacts						
C5...O2'	3.2102(3)	3.2104(3)	3.2067(9)	3.2074(9)	3.209(2)	3.212(2)
H5...O2'	2.5915(2	2.5916(2)	2.5889(6)	2.5896(7)	2.590(1)	2.593(2)
C6...O2'	3.0983(3)	3.0984(3)	3.0970(9)	3.0976(9)	3.0978(7)	3.101(2)
H6...O2'	2.3568(2)	2.3569(2)	2.3548(6)	2.3550(7)	2.356(1)	2.359(2)
C1...O4'	3.3992(4)	3.3995(4)	3.3988(9)	3.3983(10)	3.3989(5)	3.403(2)
H11...O4'	2.3184(2)	2.3185(2)	2.3175(6)	2.3175(7)	2.3180(6)	2.322(2)

Table S10. Third- and fourth-order Gram-Charlier coefficients (×10[4]) of 1-Methylluracil from VALRAY least-squares refinement. Terms > 3 esd are in bold red.

Atom	C1		C2		C4	
Model	C	D	C	D	C	D
C111	-0.009(6)	-0.015(6)	0.002(5)	0.003(6)	0.003(5)	0.012(6)
C112	0.018(8)	0.025(9)	-0.005(7)	-0.004(8)	0.011(7)	0.012(8)
C122	**-0.028(9)**	**-0.034(9)**	**0.023(7)**	**0.026(8)**	0.007(7)	0.018(8)
C133	-0.06(5)	-0.10(6)	0.02(4)	0.03(4)	0.03(4)	0.05(4)
C222	-0.002(6)	0.003(6)	-0.011(5)	-0.005(5)	-0.006(5)	-0.008(5)
C233	-0.05(5)	-0.03(6)	-0.05(4)	-0.09(4)	0.06(4)	0.06(4)
D1111		-0.0002(4)		0.0005(4)		0.0003(4)
D1112		**0.0046(7)**		0.0003(6)		-0.0001(7)
D1122		-0.0016(7)		-0.0010(6)		0.0002(6)
D1133		0.004(4)		-0.002(3)		--0.002(3)
D1222		**0.0036(7)**		-0.0001(6)		-0.0009(7)
D1233		0.014(6)		0.005(4)		0.002(5)
D2222		-0.0004(4)		-0.0008(3)		0.0007(3)
D2233		**0.014(4)**		0.003(3)		-0.002(3)
D3333		**0.08(2)**		**0.04(1)**		0.013(9)
**Atom**	**C5**		**C6**		**N1**	
Model	C	D	C	D	C	D
C111	0.003(6)	0.002(6)	0.010(5)	0.005(6)	-0.007(5)	-0.006(5)
C112	0.017(7)	0.019(7)	0.001(7)	-0.002(8)	**-0.022(7)**	**-0.025(7)**
C122	**0.023(7)**	**0.024(7)**	0.011(7)	0.011(8)	0.011(7)	0.014(7)
C133	0.10(4)	0.08(4)	-0.01(4)	0.01(4)	0.00(4)	0.01(4)
C222	0.001(5)	0.002(5)	**-0.018(5)**	**-0.019(5)**	**-0.020(4)**	**-0.026(5)**
C233	-0.04(4)	-0.05(4)	0.03(4)	0.04(4)	-0.06(4)	-0.07(4)
D1111		-0.0004(4)		0.0006(4)		-0.0002(3)
D1112		-0.0003(6)		-0.0005(7)		-0.0002(6)
D1122		-0.0009(6)		-0.0007(6)		0.0003(5)
D1133		0.001(3)		0.005(3)		0.003(3)
D1222		-0.0007(6)		-0.0016(6)		0.0006(7)
D1233		0.008(5)		0.006(5)		-0.002(4)
D2222		**-0.0010(3)**		0.0002(4)		-0.0004(3)
D2233		0.004(3)		-0.008(3)		-0.000(3)
D3333		0.02(1)		0.003(9)		0.02(1)

Atom	N3		O2		O4	
Model	C	D	C	D	C	D
C111	-0.008(5)	-0.009(6)	0.007(5)	0.003(6)	**-0.030(5)**	**-0.029(6)**
C112	0.009(7)	0.008(8)	-0.018(7)	-0.014(8)	0.008(7)	0.008(8)
C122	-0.005(7)	0.003(7)	0.011(7)	0.007(7)	-0.017(7)	-0.016(7)
C133	-0.09(4)	-0.11(4)	0.05(4)	0.05(4)	-0.05(4)	-0.03(4)
C222	**-0.015(5)**	**-0.016(5)**	**-0.026(5)**	**-0.022(5)**	-0.002(5)	-0.001(5)
C233	-0.01(4)	-0.02(4)	-0.01(4)	-0.00(4)	0.03(4)	0.04(4)
D1111		0.0003(3)		0.0006(4)		0.0004(4)
D1112		-0.0000(6)		-0.0017(7)		-0.0005(7)
D1122		-0.0004(5)		-0.0010(5)		-0.0008(5)
D1133		**0.010(3)**		0.004(3)		-0.003(3)
D1222		0.0007(6)		-0.0014(6)		-0.0002(6)
D1233		0.004(4)		-0.006(4)		0.007(4)
D2222		-0.0007(3)		-0.0001(3)		-0.0003(3)
D2233		-0.003(3)		-0.008(3)		-0.003(3)
D3333		0.024(9)		-0.019(9)		-0.003(9)
**Atom**	**H3**	**H5**	**H6**	**H11**	**H12**	
Model	C	C	C	C	C	
C111	-0.6(3)	0.2(3)	0.4(3)	**-1.6(4)**	0.3(2)	
C112	-0.3(8)	0.1(7)	0.5(7)	-0.3(8)	0.1(4)	
C122	-1.5(8)	0.3(6)	-0.2(6)	2.3(8)	-0.4(6)	
C113					**4(1)**	
C123					2(2)	
C133	2(4)	0(3)	-5(4)	**15(5)**	-7(3)	
C222	-0.6(4)	0.1(3)	**-0.9(3)**	-0.5(4)	-0.1(4)	
C223					-3(2)	
C233	-5(4)	1(3)	6(3)	3(6)	-10(4)	
C333					-6(4)	

S5. Multipole analyis and electrostatic moments

S5.1 The electroneutrality constraint.

An important indicator of the goodness of fit, in addition to those already discussed, is represented by the scale factor of the pseudo-atomic populations that define the multipolar expansion of the X-ray scattering intensities. This scale factor ensures the electroneutrality of the system and therefore assignes to the parameters of the multipolar expansion a precise physical meaning, and a role that is well above that of simple fitting parameters. A scale factor equal to one confirms an excellent choice of the mutipole model. For the four models studied,

the scale factor assumes the following values: 0.99973 (A), 0.99983 (B), 1.00018 (C), and 1.00025 (D), which correct a charge defect/excess equal to -0.018 (0.027%), -0.011 (0.017%), +0.012 (0.018%) and +0.016 (0.025%) electrons per molecule, respectively. It is evident that these values are very close to unity and the difference between them is far less than the standard deviation of 0.001 electrons per molecule. Nonetheless, an increase in values from model A through model D is well delineated and could be ascribed to the increase in the number of least-squares variables. The use of the scale factor preserves the physical meaning of the multipole expansion parameters and mitigates the consequences of a possible overfitting.

S5.2 QTAIM results

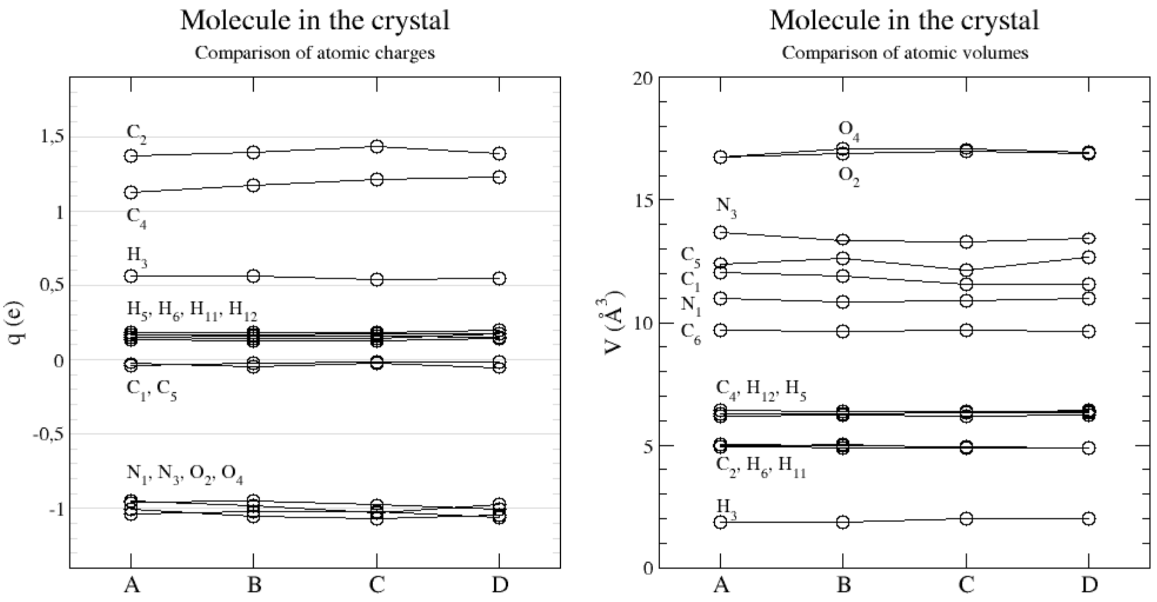

Figure S9. Comparison of QTAIM–derived atomic charges (left) and volumes (right) as a function of the multipole model (A–D). The corresponding quantities are shown as tabular entries in Table 4 (see the main text).

QTAIM results for atomic charges and volumes of a 1-methyluracil molecule rigidly extracted from the crystal are reported in Table S11.

QTAIM values of molecular Cartesian moments of both a molecule in the crystal and a molecule rigidly extracted from the crystal are compared in Table S12. These values are reported for Cartesian moment tensors expressed in their "raw" or "unabridged" form, which, being equipped with a non-null trace, has a greater content of information. For example, the trace of the quadrupole tensor is proportional to the spheropole $<r^2>$.

In both Tables, the experimental values are compared with the results of a quantum mechanical calculation. Our theoretical model is based on the B3LYP/6-311G(d,p) level of theory and uses the experimental XRD geometry. A geometry optimization was performed to obtain the wave function of a molecule in the gas phase. Periodic calculations were simulated by embedding a molecule in a field of 9000 point charges located at the atomic positions in the crystal. After the calculation was done, a new set of charges was derived and the procedure repeated with the new set of charges until convergence to a fixed set of values was reached. Calculation were done using the ORCA code.[6] Experimental and theoretical data are also compared in Figures S11 and S12.

[6] Neese, F. (2012) The ORCA program system, *Wiley Interdiscip. Rev.: Comput. Mol. Sci.*, **2012**, *2*, 73–78.

Table S11. QTAIM estimated atomic charges q (*e*) and volumes V (Å^3) for a molecule extracted from the crystal.

model	A		B		C		D		arithmetic averages		theory§	
atom	q	V	q	V	q	V	q	V	q	V	q	V
C1	-0.042	10.83	-0.035	10.83	-0.017	11.43	-0.016	10.99	-0.03(1)	11.0(3)	0.366	9.19
C2	1.369	5.74	1.394	5.68	1.437	5.59	1.388	5.60	1.40(3)	5.65(6)	1.768	5.14
C4	1.123	6.66	1.171	6.67	1.210	6.82	1.229	6.60	1.18(4)	6.69(8)	1.327	6.45
C5	-0.032	12.16	-0.051	12.24	-0.027	12.08	-0.066	12.19	-0.04(2)	12.17(6)	-0.002	12.24
C6	0.149	11.41	0.147	11.47	0.148	11.08	0.174	10.83	0.15(1)	11.2(3)	0.402	10.31
N1	-0.965	11.91	-0.955	11.97	-0.983	11.90	-1.008	11.87	-0.98(2)	11.91(4)	-1.179	11.80
N3	-1.038	13.97	-1.021	13.73	-1.020	13.80	-1.059	13.89	-1.03(2)	13.85(9)	-1.141	14.53
O2	-0.948	17.03	-0.981	17.28	-1.033	17.41	-0.976	17.41	-0.98(3)	17.3(2)	-1.127	19.04
O4	-0.985	17.46	-1.034	17.91	-1.052	18.18	-1.027	17.87	-1.02(3)	17.9(3)	-1.092	19.79
H3	0.548	3.28	0.546	3.29	0.518	3.58	0.527	3.63	0.54(1)	3.5(2)	0.429	4.32
H5	0.137	5.61	0.132	5.88	0.132	5.80	0.142	5.63	0.136(4)	5.7(1)	0.067	6.65
H6	0.185	5.24	0.189	5.30	0.187	5.32	0.199	5.39	0.190(5)	5.31(5)	0.060	6.85
H11	0.168	5.24	0.177	5.10	0.183	4.98	0.180	5.17	0.177(6)	5.1(1)	0.021	6.66
H12	0.166	5.48	0.161	5.38	0.160	5.35	0.155	5.57	0.161(4)	5.45(9)	0.051	6.51
H12	0.166	5.48	0.161	5.38	0.160	5.35	0.155	5.57	0.161(4)	5.45(9)	0.051	6.51
∑	0	137.47	0	138.09	0	138.67	0	138.23	0	138.1(4)	0	145.97

§ Computed from the B3LYP/6-311G(d,p) wave function of the isolated molecule at crystal geometry obtained with the ORCA program.

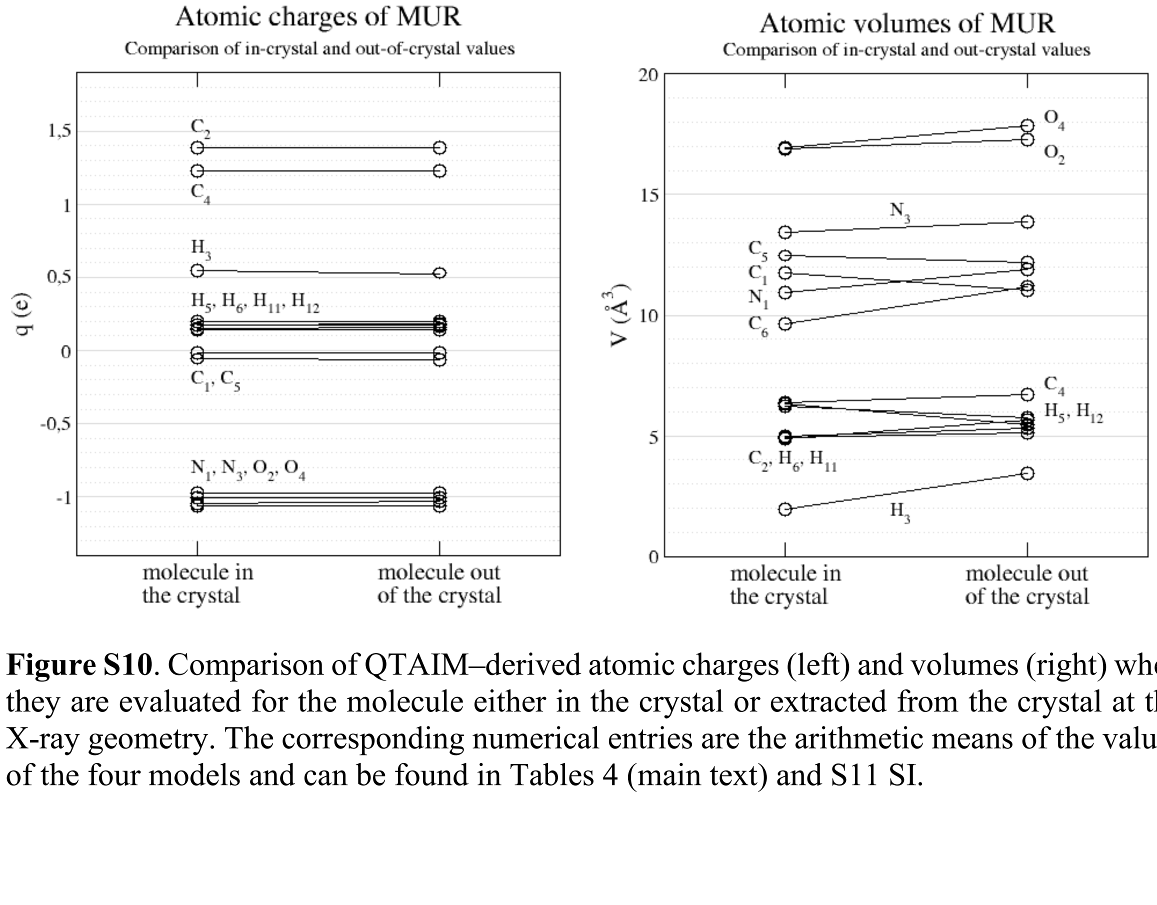

Figure S10. Comparison of QTAIM–derived atomic charges (left) and volumes (right) when they are evaluated for the molecule either in the crystal or extracted from the crystal at the X-ray geometry. The corresponding numerical entries are the arithmetic means of the values of the four models and can be found in Tables 4 (main text) and S11 SI.

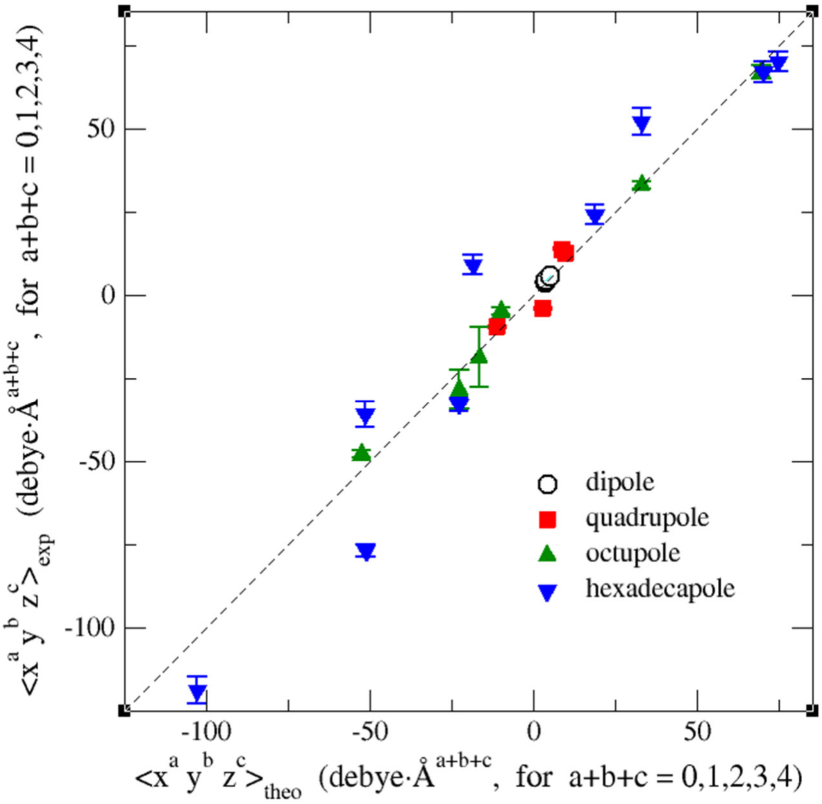

Figure S11. Comparison of experimental *vs* theoretical traceless Cartesian moments of the 1-methyluracil molecule in crystal. Regression lines, not shown in the figure, are:
$m^{(1)}_{exp} = 1.2(3)\ m^{(1)}_{theo} + 0(1)$, r = 0.9691;
$m^{(2)}_{exp} = 1.1(4)\ m^{(2)}_{theo} + 1(3)$, r = 0.9000;
$m^{(3)}_{exp} = 0.97(5)\ m^{(3)}_{theo} + 0(2)$, r = 0.9956;
$m^{(4)}_{exp} = 1.1(1)\ m^{(4)}_{theo} + 1(6)$, r = 0.9667.
Experimental values are the weighted means of the values of the four models. The standard errors of the weighted means are reported as vertical bars. The numerical values can be found in Table 5 (main text). The 45° dashed line represents perfect agreement.

Table S12. Electrostatic Unabridged Moments of the 1-Methyluracil molecule. Comparison of PAMoC results from the QTAIM DMA of the EDD for the molecule in the crystal and *extracted* from the crystal. Origin: center of mass. System of coordinates: principal axes of inertia of the molecule.

<*O*(**r**)> #	models								aritmethic averages		theory♠	
	A		B		C		D					
	crystal	isolated	crystal	isolated	crystal	isolated	crystal	isolated	crystal	isolated	crystal	isolated
<q>	0	0	0	0	0	0	0	0	0	0	0	0
<x>	3.45	3.41	3.67	3.44	3.71	3.63	3.50	3.60	3.6(1)	3.5(1)	4.31	3.35
<y>	4.07	4.47	4.27	4.62	4.11	4.47	4.07	4.42	4.13(8)	4.49(8)	4.27	3.59
|**μ**|*	5.34	5.62	5.63	5.76	5.54	5.76	5.37	5.70	5.5(1)	5.71(6)	6.07	4.91
<xx>	-54.9	-55.9	-55.1	-56.5	-53.6	-54.5	-54.8	-55.1	-54.6(6)	-55.5(8)	-65.4	-61.3
<xy>	8.1	8.2	8.4	8.4	8.1	8.0	8.1	8.3	8.2(1)	8.2(2)	8.0	6.3
<yy>	-37.8	-39.6	-37.9	-39.7	-37.5	-39.4	-37.8	-39.8	-37.8(2)	-39.6(2)	-45.8	-48.0
<zz>	-49.8	-51.7	-49.7	-51.6	-49.6	-51.6	-49.5	-51.3	-49.7(1)	-51.6(2)	-52.0	-52.0
⅓Tr ***Q*** ■	-47.5	-49.1	-47.6	-49.3	-46.9	-48.5	-47.4	-48.7	-47.4(3)	-48.9(3)	-54.4	-53.8
α ¶	20.6	20.4	21.0	20.9	20.2	19.7	20.6	19.9	20.6(3)	20.2(5)	22.2	16.1
<xxx>	56.5	54.9	57.6	53.5	54.2	52.5	51.1	52.0	54.9(2)	53(1)	53.4	44.7
<yyy>	10.5	14.6	11.6	15.8	10.9	15.5	11.1	15.1	11.0(4)	15.3(5)	6.4	1.4
<xyy>	-11.7	-10.2	-11.7	-10.3	-10.3	-9.0	-10.3	-8.7	-11.0(7)	-9.6(7)	-13.2	-15.5
<xxy>	18.1	18.8	19.0	19.3	18.2	18.6	18.1	19.0	18.4(4)	18.9(3)	18.3	14.8
<xzz>	1.6	1.8	1.9	2.0	2.0	2.1	1.9	2.2	1.9(2)	2.0(2)	-1.1	-1.2
<yzz>	-5.5	-5.8	-5.4	-5.7	-5.3	-5.5	-5.4	-5.6	-5.4(1)	-5.7(1)	-7.1	-7.3
|**β**|†	51.8	54.1	54.1	53.9	51.7	53.8	48.9	53.7	52(2)	53.9(2)	42.9	29.4
β_μ‡	47.6	50.1	50.3	50.6	48.4	50.9	45.9	50.8	48(2)	50.6(3)	40.2	25.6
<xxxx>	-912.5	-941.4	-918.9	-955.1	-898.2	-926.6	-914.1	-930.7	-911(8)	-938(11)	-1054.8	-1003.2
<yyyy>	-297.4	-322.9	-297.3	-322.8	-296.0	-322.1	-298.1	-325.8	-297.2(8)	-323(1)	-375.0	-394.8
<zzzz>	-44.2	-56.0	-44.6	-56.4	-44.4	-56.2	-44.2	-55.4	-44.4(2)	-56.0(4)	-56.0	-55.8
<xxxy>	10.7	12.6	10.7	11.9	12.3	14.31	12.8	17.5	11.6(9)	14(2)	0.8	-4.8
<yyyx>	41.8	38.7	42.7	40.1	40.3	36.6	40.9	38.2	41.4(9)	38(1)	36.3	24.0
<xxyy>	-184.2	-195.6	-183.6	-194.0	-180.8	-191.8	-182.0	-192.7	-183(1)	-194(1)	-220.9	-222.3
<xxzz>	-138.9	-146.0	-138.2	-145.6	-137.4	-144.9	-138.3	-144.9	-138.2(5)	-145.4(5)	-158.6	-155.9
<yyzz>	-77.2	-82.4	-76.7	-82.0	-76.6	-81.9	-76.7	-82.1	-76.8(2)	-82.1(2)	-86.1	-87.0
<zzxy>	-0.9	-0.2	-0.6	0.0	-0.7	-0.3	-0.5	0.2	-0.7(2)	-0.7(2)	-2.1	-2.9
⅓Tr ***H***§	-684.9	-722.8	-685.9	-725.8	-676.1	-714.0	-683.5	-717.0	-683(4)	-720(5)	-805.7	-794.7

Expectation value of the moment operator O(r), that is 1 for charge, r for dipole moment, **r**⊗**r** for quadrupole moment, etc. Units are electron for charge (ℓ = 0), debye for dipole moment (ℓ = 1), debye·Å or buckingham for quadrupole moment (ℓ = 2), and debye·$Å^{ℓ}$ for higher order moments (ℓ ≥ 3).

♠ Computed from the B3LYP/6-311G(d,p) wave function obtained with the ORCA programi (see text).

* Magnitude of the dipole moment: $|\boldsymbol{\mu}| = (\langle x\rangle^2 + \langle y\rangle^2 + \langle z\rangle^2)^{-½}$.

■ Isotropic quadrupole value or quadrupole mean value: ⅓ (<xx> + <yy> + <zz>)

¶ Quadrupole anisotropy (see similar note in Table 7).

† $\beta_i = \Sigma_j$ <ijj>, for i, j = x, y, z, and $|\boldsymbol{\beta}| = (\Sigma_j \beta_i^2)^{-½}$.

‡ $\beta_\mu = (\Sigma_i \beta_i \mu_i)/|\boldsymbol{\mu}|$, for i, j = x, y, z.

§ Isotropic hexadecapole value or hexadecapole mean value: ⅓ Σi Σj≠i <iijj>, for i, j = x, y, z.

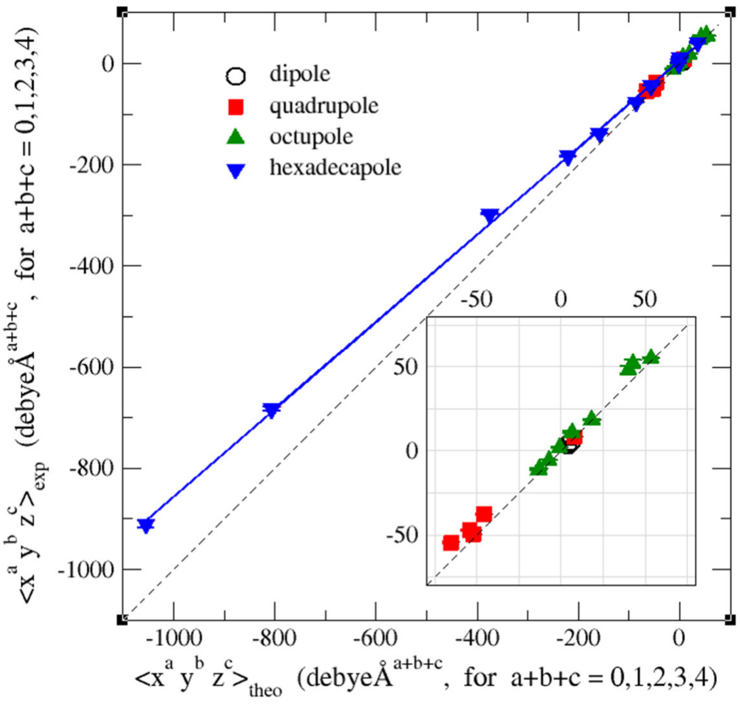

Figure S12. Comparison of experimental *vs* theoretical unabridged Cartesian moments of the 1-methyluracil molecule in crystal (numerical entries in Table S12). Arithmetic averages from the 4 multipole models are employed as the least biased experimental estimates. Regression lines are described by the following equations:
$m^{(1)}_{exp} = 0.9(3)\ m^{(1)}_{theo} + 0(1)$, r = 0.9132;
$m^{(2)}_{exp} = 0.88(5)\ m^{(2)}_{theo} + 1(3)$, r = 0.9942;
$m^{(3)}_{exp} = 1.06(5)\ m^{(3)}_{theo} + 3(1)$, r = 0.9943;
$m^{(4)}_{exp} = 0.862(8)\ m^{(4)}_{theo} + 6(4)$, r = 0.9996.
Only the regression line for the fourth order moments is shown in the figure. The standard uncertainties of the experimental values are reported as vertical bars. Dashed lines are the 45°-lines of perfect agreement. The inset is an enlargement of the upper right corner of the main graph.

S5.3 Stewart's unabridged Cartesian moment tensors.

Stewart atoms are obtained from the least-squares structure refinement in the form of spherical harmonics multipole moments and can be easily transformed into the form of traceless Cartesian moment tensors by simple linear relations. In addition, Stewart's unabridged Cartesian moment tensors can be obtained by calculating the expectation value of each component moment by volume integration over all space. Atomic volumes, and hence molecular volumes, can be estimated as well by volume integration. Molecular unabridged moments obtained from Stewart's DMA are reported in Table S13 and compared graphically with those obtained from the QTAIM DMA in Figure 6 (see the main text). The molecular volume obtained as the sum of Stewart atom volumes (see last row of Table S13) is more than two times greater than that obtained by adding QTAIM atomic volumes. This is a consequence of the fuzzy character of Stewart atoms and requires a redefinition of the isodensity value used for the determination of atomic volumes.

Table S13. Electrostatic Unabridged Moments of the 1-Methyluracil molecule removed from the crystal, obtained from the unabridged Cartesian moments of Stewart's atoms. Origin: center of mass. System of coordinates: principal axes of inertia of the molecule. Molecular volumes are reported in the last row. The arithmetic mean of the values obtained for the four models is reported in the sixth column.

<*O*(**r**)> #	Models				Stewart's DMA	QTAIM DMA♠
	A	B	C	D		
<q>	0	0	0	0	**0**	0
<x>	3.49	3.72	3.83	3.58	**3.7(1)**	3.5(1)
<y>	4.47	4.66	4.44	4.43	**4.50(9)**	4.49(8)
|**μ**|*	5.67	5.97	5.87	5.70	**5.8(1)**	5.71(6)
<xx>	-55.4	-56.6	-54.3	-55.2	**-55.4(8)**	-55.5(8)
<xy>	8.2	8.5	8.3	8.4	**8.4(1)**	8.2(2)
<yy>	-39.6	-39.7	-39.6	-39.8	**-39.7(1)**	-39.6(2)
<zz>	-51.7	-51.6	-51.6	-51.3	**-51.6(2)**	-51.6(2)
⅓Tr ***Q*** ▪	-48.9	-49.0	-48.5	-48.8	**-48.8(2)**	-48.9(3)
α ¶	20.1	20.6	19.8	20.1	**20.2(3)**	20.2(5)
<xxx>	56.9	57.9	54.6	51.1	**55(3)**	53(1)
<yyy>	14.5	15.8	14.9	15.0	**15.1(5)**	15.3(5)
<xyy>	-10.4	-10.3	-8.5	-8.7	**-9.5(9)**	-9.6(7)
<xxy>	18.8	19.8	18.7	18.9	**19.1(4)**	18.9(3)
<xzz>	1.8	2.1	2.3	2.2	**2.1(2)**	2.0(2)
<yzz>	-5.8	-5.7	-5.6	-5.6	**-5.7(1)**	-5.7(1)
|**β**|†	55.6	58.0	56.0	52.9	**56(2)**	53.9(2)
β_μ‡	51.5	54.4	52.8	50.1	**52(2)**	50.6(3)
<xxxx>	-932.6	-939.2	-921.1	-933.4	**-932(6)**	-938(11)
<yyyy>	-322.5	-322.5	-323.4	-326.0	**-324(1)**	-323(1)
<zzzz>	-55.8	-56.2	-56.1	-55.2	**-55.8(4)**	-56.0(4)
<xxxy>	12.7	13.5	16.0	17.1	**15(2)**	14(2)
<yyyx>	38.4	40.2	38.0	38.5	**38.8(8)**	38(1)
<xxyy>	-195.8	-193.8	-192.0	-192.5	**-194(1)**	-194(1)
<xxzz>	-145.8	-145.0	-144.5	-144.8	**-145.0(5)**	-145.4(5)
<yyzz>	-82.3	-81.9	-81.9	-81.9	**-82.0(2)**	-82.1(2)
<zzxy>	-0.2	0.0	0.1	0.2	**0.0(2)**	-0.7(2)
⅓Tr ***H***§	-719.5	-719.8	-712.4	-717.7	**-717(3)**	-720(5)
volume	296.97	296.45	296.04	295.10	**296.1(8)**	138.1(4) ♠

Expectation value of the moment operator O(r), that is 1 for charge, r for dipole moment, **r**⊗**r** for quadrupole moment, etc. Units are electron for charge (ℓ = 0), debye for dipole moment (ℓ = 1), debye·Å or buckingham for quadrupole moment (ℓ = 2), and debye·Å$^{\ell}$ for higher order moments (ℓ ≥ 3).

♠ Data are reported from Table S10.

* Magnitude of the dipole moment: $|\boldsymbol{\mu}| = (\langle x\rangle^2 + \langle y\rangle^2 + \langle z\rangle^2)^{-½}$.

■ Isotropic quadrupole value or quadrupole mean value: ⅓ (<xx> + <yy> + <zz>)

¶ Quadrupole anisotropy (see similar note in Table 7).

† $\beta_i = \Sigma_j \langle ijj\rangle$, for i, j = x, y, z, and $|\boldsymbol{\beta}| = (\Sigma_j \beta_i^2)^{-½}$.

‡ $\beta_\mu = (\Sigma_i \beta_i \mu_i)/|\boldsymbol{\mu}|$, for i, j = x, y, z.

§ Isotropic hexadecapole value or hexadecapole mean value: ⅓ Σi Σj≠i <iijj>, for i, j = x, y, z.

♠ Value taken from Table S12.
