# Supplementary material for: Anharmonic Thermal Motion Modelling in the Experimental XRD Charge Density Determination of 1-Methyluracil at T = 23 K
Source: Molecules. 2021 May 21;26(11):3075. doi: 10.3390/molecules26113075 (PMC8196607; doi:10.3390/molecules26113075)

checkCIF/PLATON report

Structure factors have been supplied for datablock(s) I

THIS REPORT IS FOR GUIDANCE ONLY. IF USED AS PART OF A REVIEW PROCEDURE FOR PUBLICATION, IT SHOULD NOT REPLACE THE EXPERTISE OF AN EXPERIENCED CRYSTALLOGRAPHIC REFEREE.

No syntax errors found. CIF dictionary Interpreting this report

Datablock: I

```
Bond precision:  C-C = 0.0008 A          Wavelength=0.71073

Cell:              a=13.208(1)      b=13.164(1)      c=6.1620(6)
                   alpha=90         beta=90          gamma=90
Temperature:       23 K

                Calculated                  Reported
Volume          1071.39(16)                 1071.4(2)
Space group     I b a m                     I b a m
Hall group      -I 2 2c                     -I_2_2c
Moiety formula  C5 H6 N2 O2                 C5 H6 N2 O2
Sum formula     C5 H6 N2 O2                 C5 H6 N2 O2
Mr              126.12                      126.12
Dx,g cm-3       1.564                       1.564
Z               8                           8
Mu (mm-1)       0.124                       0.124
F000            528.0                       528.0
F000'           528.27
h,k,lmax        30,30,14                    30,30,14
Nref            3569                        3568
Tmin,Tmax       0.959,0.976
Tmin'           0.959

Correction method= Not given

Data completeness= 1.000          Theta(max)= 54.554

R(reflections)= 0.0237( 2783)     wR2(reflections)= 0.0314( 3568)

S = 0.947                  Npar= 393
```

```
The following ALERTS were generated. Each ALERT has the format
       test-name_ALERT_alert-type_alert-level.
Click on the hyperlinks for more details of the test.
```

Alert level C

```
CELLK01_ALERT_1_C  Check that the cell measurement temperature is in Kelvin.
            Value of measurement temperature given =    23.000
```

Author Response: We confirm that the cell measurement temperature is in Kelvin.

```
PLAT088_ALERT_3_C Poor Data / Parameter Ratio ....................       9.08 Note
```

Author Response: This structural model comes from a multipole refinement against a very accurate dataset collected at 23 K up to 109 deg in 2\J. The data-to-parameter ratio is reasonable.

```
PLAT218_ALERT_3_C Constrained U(ij) Components(s) for H12        .          6 Check
```

Author Response: Anisotropic U(ij) components of all the hydrogen atoms were taken from Roversi, P.; Destro, R. Approximate anisotropic displacement parameters for H atoms in molecular crystals. Chem. Phys. Lett. 2004, 386, 472--478, Table B, and never refined. More in detail, they were estimated by adding IR-expected vibrational amplitudes to the molecular rigid-body motion. U(ij) parameters of hydrogen atoms were then kept fixed in the subsequent multipolar refinement procedure.

```
PLAT250_ALERT_2_C Large U3/U1 Ratio for Average U(i,j) Tensor ....        2.1 Note
```

Author Response: This is due to a large translation component of the molecular rigid body motion in the direction perpendicular to the main molecular plane. Considering the quality of the present structural determination (very low temperature, aspherical features of the charge density accounted for), we believe that this reflects true dynamic properties of the lattice.

```
PLAT353_ALERT_3_C Long   N-H (N0.87,N1.01A)  N3       - H3       .       1.04 Ang.
```

Author Response: The position of hydrogen atoms was taken from a previous neutron diffraction study (McMullan, R.K.; Craven, B.M. Crystal structure of 1-methyluracil from neutron diffraction at 15, 60 and 123 K. Acta Crystallogr. Sect. B Struct. Sci. 1989, 45, 270--276) and never refined.

```
PLAT977_ALERT_2_C Check Negative Difference Density on H11             -0.32 eA-3
```

Author Response: This problem arises as PLATON uses spherical atomic form factors to predict structure factor amplitudes. When the multipole model is employed, no negative residuals are indeed detectable in the neighborhood of H11. See for example Figure 3 in the main text.

Alert level G

```
PLAT007_ALERT_5_G Number of Unrefined Donor-H Atoms ..............          1 Report
PLAT152_ALERT_1_G The Supplied and Calc. Volume s.u. Differ by ...         -4 Units
PLAT380_ALERT_4_G Incorrectly? Oriented X(sp2)-Methyl Moiety .....         C1 Check
PLAT808_ALERT_5_G No Parseable SHELXL Style Weighting Scheme Found     Please Check
PLAT881_ALERT_1_G No Datum for _diffrn_reflns_av_R_equivalents ...     Please Do !
PLAT912_ALERT_4_G Missing # of FCF Reflections Above STh/L=  0.600          1 Note
PLAT929_ALERT_5_G No Weight Pars,Obs and Calc R1,wR2,S not Checked          ! Info
PLAT941_ALERT_3_G Average HKL Measurement Multiplicity ...........        1.0 Low
PLAT978_ALERT_2_G Number C-C Bonds with Positive Residual Density.          2 Info
```

---

   0 **ALERT level A** = Most likely a serious problem - resolve or explain
   0 **ALERT level B** = A potentially serious problem, consider carefully
   6 **ALERT level C** = Check. Ensure it is not caused by an omission or oversight
   9 **ALERT level G** = General information/check it is not something unexpected

   3 ALERT type 1 CIF construction/syntax error, inconsistent or missing data
   3 ALERT type 2 Indicator that the structure model may be wrong or deficient
   4 ALERT type 3 Indicator that the structure quality may be low
   2 ALERT type 4 Improvement, methodology, query or suggestion
   3 ALERT type 5 Informative message, check

---

checkCIF publication errors

Alert level G

PUBL017_ALERT_1_G The _publ_section_references section is missing or
            empty.

---

   0 **ALERT level A** = Data missing that is essential or data in wrong format
   1 **ALERT level G** = General alerts. Data that may be required is missing

Publication of your CIF

You should attempt to resolve as many as possible of the alerts in all categories. Often the minor alerts point to easily fixed oversights, errors and omissions in your CIF or refinement strategy, so attention to these fine details can be worthwhile. In order to resolve some of the more serious problems it may be necessary to carry out additional measurements or structure refinements. However, the nature of your study may justify the reported deviations from journal submission requirements and the more serious of these should be commented upon in the discussion or experimental section of a paper or in the "special_details" fields of the CIF. *checkCIF* was carefully designed to identify outliers and unusual parameters, but every test has its limitations and alerts that are not important in a particular case may appear. Conversely, the absence of alerts does not guarantee there are no aspects of the results needing attention. It is up to the individual to critically assess their own results and, if necessary, seek expert advice.

If you wish to submit your CIF for publication in Acta Crystallographica Section C or E, you should upload your CIF via the web. If you wish to submit your CIF for publication in IUCrData you should upload your CIF via the web. If your CIF is to form part of a submission to another IUCr journal, you will be asked, either during electronic submission or by the Co-editor handling your paper, to upload your CIF via our web site.

PLATON version of 22/03/2021; check.def file version of 19/03/2021

Datablock I - ellipsoid plot

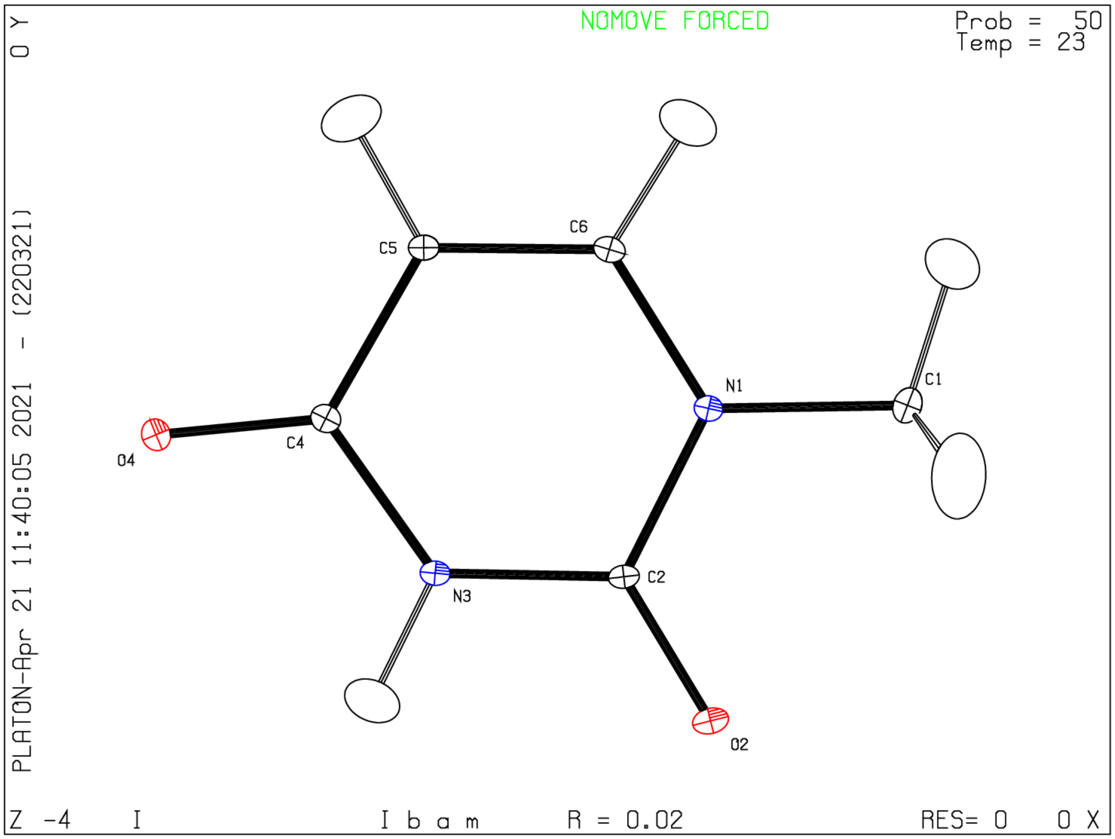

Supplement: Supplementary file 1 [file MUR23_checkcif.pdf › MUR23_checkcif.pdf]
